# QUADrATiC: scalable gene expression connectivity mapping for repurposing FDA-approved therapeutics

**DOI:** 10.1186/s12859-016-1062-1

**Published:** 2016-05-04

**Authors:** Paul G. O’Reilly, Qing Wen, Peter Bankhead, Philip D. Dunne, Darragh G. McArt, Suzanne McPherson, Peter W. Hamilton, Ken I. Mills, Shu-Dong Zhang

**Affiliations:** Centre for Cancer Research and Cell Biology, Queen’s University Belfast, Belfast, BT9 7AE UK; Northern Ireland Centre for Stratified Medicine, University of Ulster, C-TRIC Building, Altnagelvin Hospital campus, Glenshane Road, Derry/Londonderry, BT47 6SB UK

**Keywords:** Connectivity mapping, Multicore programming, Big data, Repurposing, Drug discovery, Bioinformatics, Computational biology

## Abstract

**Background:**

Gene expression connectivity mapping has proven to be a powerful and flexible tool for research. Its application has been shown in a broad range of research topics, most commonly as a means of identifying potential small molecule compounds, which may be further investigated as candidates for repurposing to treat diseases. The public release of voluminous data from the Library of Integrated Cellular Signatures (LINCS) programme further enhanced the utilities and potentials of gene expression connectivity mapping in biomedicine.

**Results:**

We describe QUADrATiC (http://go.qub.ac.uk/QUADrATiC), a user-friendly tool for the exploration of gene expression connectivity on the subset of the LINCS data set corresponding to FDA-approved small molecule compounds. It enables the identification of compounds for repurposing therapeutic potentials. The software is designed to cope with the increased volume of data over existing tools, by taking advantage of multicore computing architectures to provide a scalable solution, which may be installed and operated on a range of computers, from laptops to servers. This scalability is provided by the use of the modern concurrent programming paradigm provided by the Akka framework. The QUADrATiC Graphical User Interface (GUI) has been developed using advanced Javascript frameworks, providing novel visualization capabilities for further analysis of connections. There is also a web services interface, allowing integration with other programs or scripts.

**Conclusions:**

QUADrATiC has been shown to provide an improvement over existing connectivity map software, in terms of scope (based on the LINCS data set), applicability (using FDA-approved compounds), usability and speed. It offers potential to biological researchers to analyze transcriptional data and generate potential therapeutics for focussed study in the lab. QUADrATiC represents a step change in the process of investigating gene expression connectivity and provides more biologically-relevant results than previous alternative solutions.

**Electronic supplementary material:**

The online version of this article (doi:10.1186/s12859-016-1062-1) contains supplementary material, which is available to authorized users.

## Background

### Connectivity mapping and LINCS

Gene expression connectivity mapping has proven, since its introduction [1], to be a powerful and flexible tool for research. Its application has been shown in a broad range of research topics, most commonly as a means of identifying potential small molecule compounds, which may be further investigated as candidates for repurposing to treat diseases [2, 3]. Connectivity mapping has been demonstrated for transcriptomic signatures derived from microarray and Next Generation Sequencing (NGS) platforms [4], and, as such, holds promise as an important downstream analysis. The CMap database [5] consists of 6100 gene expressions for 1309 perturbagens over 5 cell lines. However, in 2014 the Broad Institute released the Library of Integrated Network-based Cellular Signatures (LINCS) covers a vastly broader range of cell types (18) and perturbagens (20413 small molecules and 22119 genetic perturbagens), resulting in approximately 1.3M experimental reference profiles, with each containing the measured expression of the 978 landmark genes, along with the inferred expression (based on a linear model) of a further 21305 probes (to make up the entire Affymetrix HGU133A microarray probe set). With this larger database, it is necessary to develop a framework which scales beyond the implementations developed previously for the CMap data, and QUADrATiC (QUB Accelerated Drug And Transcriptomic Connectivity) is one such software package. It makes use of advanced Big Data technologies to parallelize the discovery of connections from gene expression signatures and small-molecule compounds, producing an increase in performance of at least an order of magnitude. It is also flexible in deployment, and is capable of being deployed and operated on a range of platforms and operating systems. QUADrATiC is freely-available from (http://go.qub.ac.uk/QUADrATiC) for non-commercial use.

### Connectivity mapping concept and algorithms

The basic concept of connectivity mapping is a simple one - namely that of using the transcriptomic profile of differential gene expression as a proxy for the molecular state of cells. A connection is considered as a measure of the similarity, or difference, between the state observed in a set of experiments (application of small molecule compounds to cell- lines) and that of a condition under study (e.g. disease vs control). Since the original connectivity mapping paper [1], and following the availability of the associated original dataset through the CMap platform, other researchers have been motivated to develop and implement connectivity mapping algorithms in order to improve upon the Kolmogorov-Smirnov non-parametric score implemented in CMap [6]. One popular alternative to the standard CMap software is sscMap [7], a novel rank-based algorithm, which has overcome some of the perceived disadvantages of the original CMap algorithm. Its ability to provide estimated *p*-values for connections at individual instance or treatment set level, coupled with its demonstrated specificity, has resulted in its successful application to a number of biological problems [8–10]. The data set used by the sscMap software is based on the CMap data, and likewise consists of 6100 reference profiles. The relatively small number of profiles makes it feasible for the software to be used on a wide variety of computers, from laptops to higher- specification desktop machines, with reasonable execution times (minutes or hours).

### Multicore CPU technology

Because of its use of many independent calculations (per reference profile and per signature) sscMap, in common with many bioinformatic algorithms such as bootstrapping, lends itself to parallel implementation. Such algorithms are known as ’embarrassingly parallel’. Indeed, previous work has shown that parallel implementation on concurrent processing platforms such as General Purpose Graphics Processing Units (GPGPU) [11] can provide great performance advantages in the calculation of connection strengths and *p*-value estimates. In recent years, other options for parallel concurrent implementation of algorithms in software have come to the fore, including the use of Field-Programmable Gate Arrays (FPGA) and, as applied here, multi-core CPU architectures from Intel and other suppliers [12]. With the advent of processors containing multiple cores and utilizing ’hyperthreading’ technology, it has become possible to take advantage of these capabilities using standard programming languages and frameworks, without the specialized implementations required for FPGA and GPGPU.

### Software models for concurrency

The most common model of software concurrency over the past 20 years has been to use threads. However, complications with the model of threads (due to data corruption and locking issues with shared data across thread contexts) has led to slower adoption and much software tends to be still written for serial execution [13]. An alternative abstraction has recently become of interest within the community and has been increasingly adopted. This paradigm implements software in independent units of work which have their own state, communicate with other units of work using messages and have no globally-shared data (unlike threads). These implementation units are called ‘Actors’, and if defined at a sufficient level of granularity, allow concurrent tasks to be distributed across multiple processing units (or cores) [14]. Thus this model of concurrency is highly suited to algorithms which can be parallelized and run on multicore processors. In addition, its programming model is a simpler abstraction than threads and allows for better separation of concerns between code for concurrency and that for the algorithms, and eases issues with locking and concurrent access to shared data [15]. One particular implementation of actors is provided within the Java Virtual Machine (JVM) by an open-source library called Akka [16, 17]. Akka is a lightweight and efficient implementation of the actor paradigm, which also provides for ’remote actors’, easily allowing deployment of software in a distributed fashion across a networked cluster of computers. These remote actors provide scope for scalability above and beyond that allowed for by traditional threads on a single machine. Akka is a popular library, in use in a wide range of software solutions, but has made little impact on bioinformatics software to-date [18]. However, given that many advanced bioinformatics algorithms have the capability to be parallelized for concurrent execution, Akka is a candidate for doing this in a simple, intuitive manner.

## Implementation

### LINCS data processing

**Fig. 1:**
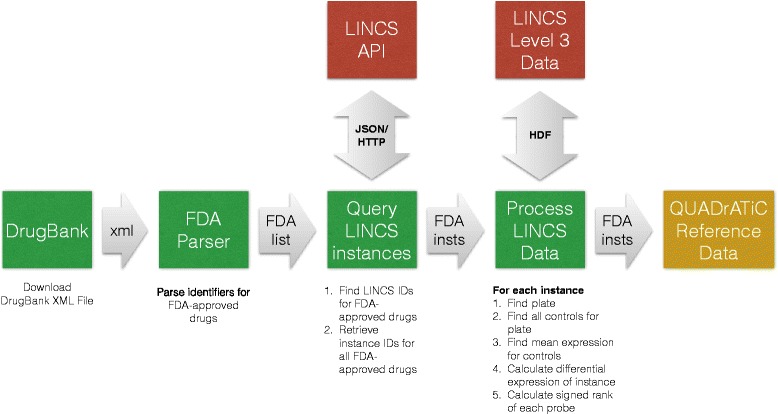
LINCS data processing flow. LINCS data processing flow to create QUADrATiC reference database

The LINCS data contains a large number of data points over a range of genetic and small-molecule compounds. This is the source of the ∼1.3M instances in the entire LINCS set. Initial analyses are often directed towards the identification of small-molecule compounds which have already been approved by the FDA for human therapeutic use, with the aim of repurposing these to treat disease [2]. Identifying and using the subset of data derived from these FDA-approved drugs has two effects - a. the production of more-readily available candidates for lab investigation, and b. the reduction in the size of the data, from 1.3M instances to 83,939, which is more easily manageable from a scalability point of view. Figure 1 shows an overview of the processing applied to the LINCS data set to extract the subset of data associated with FDA-approved drugs (as identified using the DrugBank database [19]), and make it available for use within QUADrATiC. The source data is the Level 3 data as obtained from the LINCS download site and consists of the normalized and inferred expression values for the full set of Affymetrix HGU133A probes for each experiment instance. The differential expression profiles for each instance were created by calulating the differential expression against all controls on the same plate and finding the signed rank of these.

### Improved connectivity mapping algorithm

The determination of the connection score for an instance is similar to that used by the sscMap software [7] -

$$c(\mathbf{R}, \mathbf{s})= \frac{\sum_{i=1}^{m}R(g_{i})s(g_{i})} {\sum_{i=1}^{m}N-i+1} $$

**Fig. 2:**
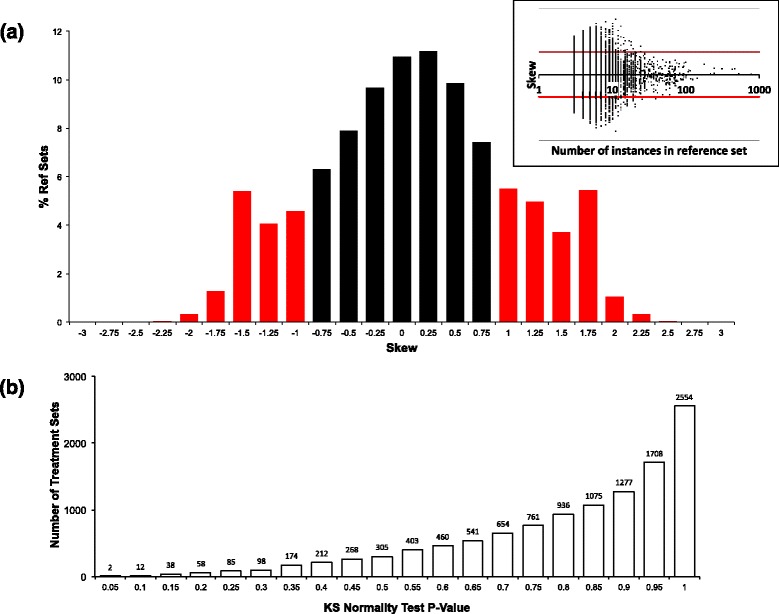
Reference set statistical landscape. **a** the variation of skew with treatment set size with, inset, the distribution of skew values for the entire set, and **b** the cumulative distribution of *p*-values for random signature score normality test

However, when grouping data into treatment sets, based on drug/concentration/cell-line etc., the scores are aggregated differently from the mean-value aggregation proposed implemented in sscMap. Presenting a random signature to the algorithm, and aggregating by reference sets grouped by perturbagen and cell line, the Pearson’s moment coefficient of skewness [20] was found. Using the threshold of gamma >1, a large proportion (more than 20 %) of treatment sets can be seen to be heavily skewed (Fig. 2(a)). This appears to be particularly associated with set sizes between 3 and 50. Given the amount of skewness evident in the scores, the median value was implemented in our framework.

### Estimation of connection *p*-values

Previous connectivity map algorithms have implemented a random sampling approach to estimation of *P*-Values for connection scores [19]. The estimate of *p*-value is calculated by comparison of the connection score for a signature and the distribution of scores produced for a large number of randomly-generated signatures. One limitation of this approach is that, in order to get resolution on the *p*-value estimates, the number of random signatures, and hence the number of connection scores calculated, must be much larger than the number of sets defined in the data. With the original data set, this is feasible (typically 10–20k signatures were enough), but with the FDA-approved data set, there are typically 10k treatment sets, and hence we require 100k random signatures to produce usable estimates. Thus, the original approach has difficulty scaling to the FDA-approved subset (or indeed to the full LINCS set). The approach taken by QUADrATiC is somewhat different. Rather than generating multiple random signatures, calculating a connection score and comparing against the set connection score individually, we propose the following:

1. Assume the random scores are normally-distributed.
  *This assumption was tested by generating scores for 50,000 random signatures and testing against the normal distribution using the Kolmogorov-Smirnov test. The distribution of p-values for this normality test are shown in Fig.*2(b), *from which it can be seen that most distributions can not be shown to be significantly different from normal.*
2. Generate a large number of connection scores per set and find the descriptive statistics (mean, standard deviation) for these.
  *Although configurable by the user, our experience is that 2000 random signatures appears to give consistent results between runs, although the option is available to increase this if required.*
3. Using the error function **erf()** to find the area under the Gaussian distibution with the mean and standard deviation calculated in step 2, calculate the probability of obtaining a connection score with absolute value greater than or equal to the absolute value of the score for the query signature.
  *This is the estimated p-value for the connection score.*

### Signature contribution fraction

Although the main application of connectivity mapping is the identification of potential drugs related to the signature presented, it is often interesting from a biological perspective to identify the importance of any particular gene in the signature to a particular connection. This may, for example help provide information as to the mechanism of action of the drug, or for grouping similar drugs. As such, QUADrATiC enables the user to investigate the effects of probes in a quantitative manner. The determination of the effect of each probe on the connection strength for a treatment set is based on a value called the Contribution Fraction (CF) of the probe. First, define the “diminished score”, for the *k*^*th*^ probe in the signature

$$c^{*}_{k}(\mathbf{R}, \mathbf{s})= \frac{\sum_{i=1, i \neq k}^{m}R(g_{i})s(g_{i})} {\sum_{i=1}^{m}N-i+1} $$

For median set scoring, as implemented by QUADrATiC, we can then define the Contribution Fraction of the *k*^*th*^ probe, *CF*_*k*_, as follows

$${CF}_{k}=1-\frac{\widetilde{c^{*}_{k}}(\mathbf{R}, \mathbf{s})}{\widetilde{c}(\mathbf{R}, \mathbf{s})} $$

i.e. the magnitude of the difference between the median score and the median diminished score (as denoted with a tilde, ∼) for the treatment set. Calculating the CF over all probes for all treatment sets can be used to investigate the influence of the signature probes on the result set. We further normalize this within the individual reference sets (where $CF^{*}_{k} = 1.0$ for the probe making the highest contribution to the connection score for that set) -

$$CF^{*}_{k}=\frac{{CF}_{k}}{\max\limits_{k}({CF}_{k})} $$

### Graphical user interface (GUI)

The GUI is developed using HTML and Javascript - providing a simple, extensible and industry- standard approach to interfacing with the user within a modern browser. The choice of javascript allows for easy use of the Bootstrap framework [21], and d3 visualisation library, [22], to provide a modern, simple interface. The GUI was designed around a simple linear workflow, which is shown, along with screenshots for the different stages of the analysis, in Fig. 3. There are six screens available to the user (Additional file 1 provides a full User Manual for the operation of QUADrATiC) -

1. Define Signatures

**Fig. 3:**
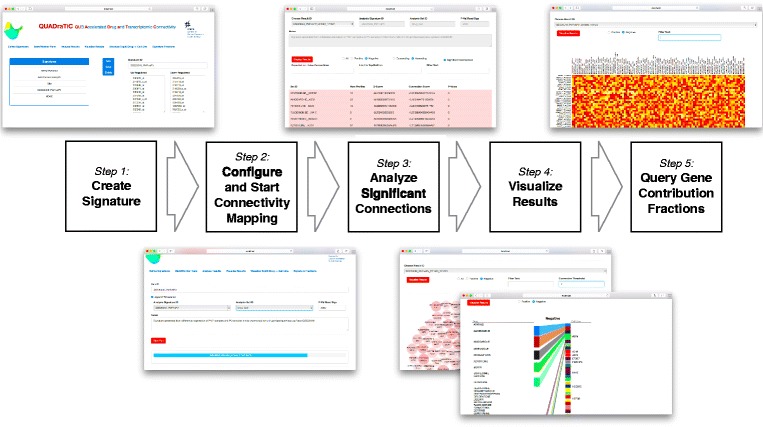
QUADrATiC Workflow and GUI. Step 1: the query signature is defined as a pair of lists of up and down-regulated Affymetrix HG-U133A probe identifiers. Step 2: an analysis run is defined by choosing the query signature, the treatment set type (grouped by drug, or by drug and cell line), and the number of random signatures to be used to estimate the *p*-value of the connections; the progress bar updates every 30 seconds. Step 3: View the table of significant connections. Step 4: Summary visualizations are available as a bubble chart representing the top connections, or a summary of drugs and cell lines (for treatment sets defined by drug and cell line). Step 5: View a heatmap showing the relative contribution of each probe in the signature
  The user can define, save, edit and delete signatures as lists of up and down-regulated Affymetrix HGU133A probe IDs.
2. Start/Monitor Runs
  The user can enter an identifier for an analysis, choose the signature (as defined in 1), the treatment sets to use (grouping by drug across all cell lines or, more usually, by drug and cell line), and set the number of random signatures to be used to estimate the *p*-value (usually 2000). The analysis can then be started and its progress monitored.
3. Analyze Results
  The user can view the detailed results of an analysis and perform simple filtering (significant/all, positive/negative/all, simple text filtering) and ordering (ascending/descending Z-Score).
4. Visualize Results
  This presents a bubble plot of the significant connections, with simple filtering options.
5. Visualize Top30 Drug $\rightarrow $ Cell Line
  This is a dynamic and interactive visualization (for treatment sets defined by drug and cell line) of the top 30 connections (negative or positive), showing relationships between the drugs and cell lines.
6. Signature Fractions
  This provides a heat map view of the normalized Connection Fractions for up to top 100 connections, matching the specified criteria. The data may be sorted by column (alphabetically) or row (by median value). Row sorting, in particular, is useful when viewing the subset of connections for a particular drug across multiple cell lines, as it can provide further information as to which genes in the signature are affected by the action of that drug. A spreadsheet-readable Comma Separated Variable (CSV) file is also produced for the data in the displayed heatmap, and may be downloaded through the browser.

### QUADrATiC server

The server component is a multicore processor-enabled parallel implementation of the algorithm, capable of handling the large quantities of data associated with the LINCS data set. It provides a web service interface using Hypertext Transfer Protocol (HTTP), which is used by the GUI and also capable of being used to integrate to other software and/or scripts.

#### Parallel implementation

**Fig. 4:**
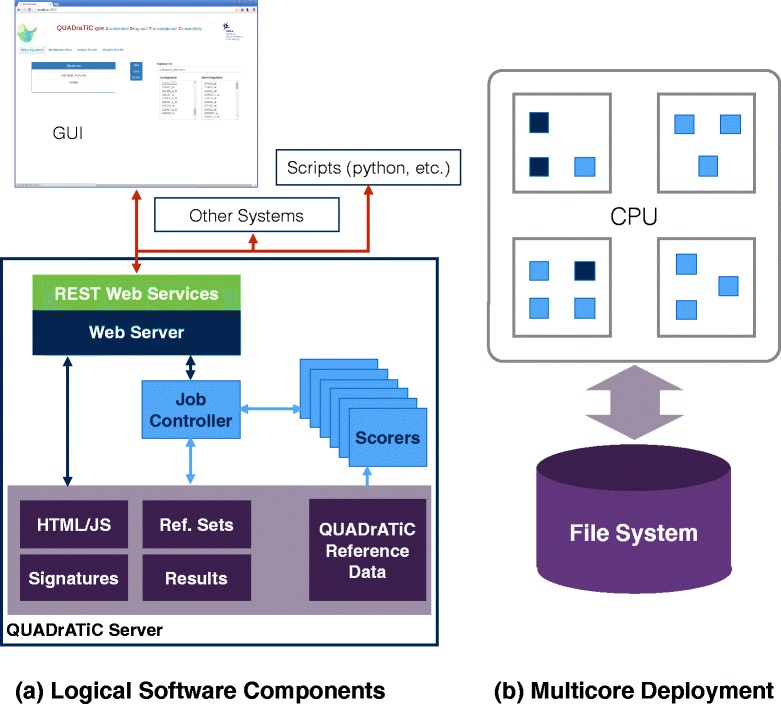
QUADrATiC Software Design. The software architecture of QUADrATiC, showing (**a**) the main software and storage logical components, and (**b**) an illustration of how these components are distributed among the cores of a multicore processor when the software runs

In the QUADrATiC implementation, the main work is carried out by two types of actors - a single Job Controller actor and multiple Scorer actors, which are scheduled across multiple cores by the Akka scheduler running in the JVM. Figure 4 shows an overview of the main interactions between these actors, and a high-level statement of the responsibilities of each. It is the decomposition into these actors that allows multiple cores to be exercised in parallel during the connectivity map calculations.

#### Web server

The implementation of the software as a web server, rather than a standalone executable, has advantages in a number of areas. One is that it allows greater flexibility in the choice of GUI technologies which may be used - including the flexibility of rendering the GUI within any modern web browser, using one of the many powerful and flexible Javascript frameworks widely available. Having this separation between the GUI and the main logic/computation carried out in the web server, also gives options in deploying the software - it may be installed solely on a desktop or laptop PC, or it may be deployed on a more powerful server and accessed by the user via the browser on their own local desktop or laptop. In addition, since the browser- based interface makes use of a standard, defined, HTTP-based Application Program Interface (API), this API may also be used to interact with and drive the server using other methods, such as scripts, or for machine-machine (M2M) integration within a larger genomics processing pipeline.

#### RESTful API

RESTful Web Services, [23], is a design methodology used widely in, but not limited to, the design and implementation of HTTP- based APIs (for GUI and M2M integration). It represents the system as a set of ’resources’, each of which is addressed using a standard HTTP Uniform Resource Identifier (URI) and can be acted upon using the HTTP ’verbs’ - GET, PUT, POST and DELETE. In our design, interactions with these resources use the common JSON format for data, and each resource encapsulates data which is represented in JSON form. The full API specification is provided in the Additional file 2.

## Results

### Performance and scalability

**Fig. 5:**
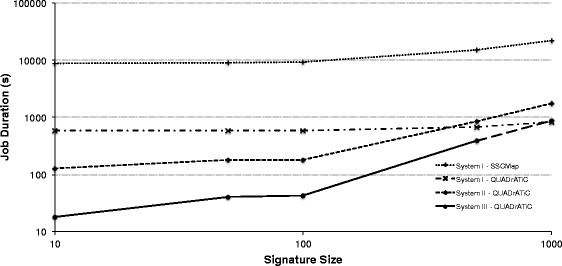
QUADrATiC Performance Test Results. Duration of time taken for QUADrATiC to complete the connectivity map analysis for four software/server configurations - System I (desktop PC with hard disk storage) using sscMap and QUADrATiC, System II (a laptop with solid state storage) using QUADrATiC, and System III (a HPC server with network-attached hard disk storage) using QUADrATiC. QUADrATiC is around one order of magnitude faster than the equivalent sscMap implementation, and, for smaller signatures, benefits from the faster file loading speeds of solid state storage

One of the major advantages of QUADrATiC is in performance, over existing standalone implementations. In order to demonstrate this, a series of performance tests were carried out on two systems, as specified in Table 1. A set of randomly-generated signatures of increasing length were created, and run on the two systems, with the same signatures being run on a comparable connectivity map application [7], which was configured to use the same LINCS subset as QUADrATiC. Figure 5 shows the variation of performance of QUADrATiC for increasing signature length on the two test systems, and comparing to the baseline performance. These performance results show that QUADrATiC offers substantially improved performance over the existing software, being an order of magnitude faster for the same size of signature queried. This is true for both test systems. The detailed performance results show evidence of the difference between the two frameworks, and in so doing, provide insights into how the software might be best deployed. For small signature sizes, the CPU load is small compared to the time required to load the reference profiles from storage. System II, having the advantage of using a Solid-State Drive (SSD) has an appreciable performance advantage over system I, since SSD storage typically has much faster read/write performance than Hard Disk Drives (HDD). As signature sizes increase, the amount of CPU power required to calculate the connection strengths increases appropriately, and the ability of the system to carry out these calculations in parallel on more powerful cores becomes more important. System I tends to perform better than system II for larger signatures, given its greater scalability in terms of cores. As evidenced in these performance figures, it can be proposed that the best system configuration for deploying QUADrATiC to its best advantage would have aspects of the two test systems, i.e. an SSD storage for the reference profiles like System II and many cores and larger RAM like System I. However, QUADrATiC can be configured to limit threads and actors to allow it to run on lower-specification systems, such as laptops or tablets, which is an increasingly important consideration in modern translational research.

**Table 1:** Systems used for performance testing

	System I	System II	System III
Manufacturer	Dell	Apple	Dell
CPU	Intel Core i7	Intel Core i7	Intel E5645
	3.7 GHz	1.7 GHz	2.4 GHz
	4 Cores	2 Cores	6 cores
	Hyperthreading ON	Hyperthreading ON	Hyperthreading ON
Cache	10 MB L3	4 MB L3	12 MB L3
	256 KB (per core) L2	256 KB (per core) L2	256 KB (per core) L2
RAM	16 GB	8 GB	24 GB
	1866 MHz	1600 MHz	1333 MHz
	DDR3	DDR3	DDR3
Storage	3.5inch Serial ATA (7,200 Rpm)	Apple SM0512F Media	Dell MD1000 SATA NFS
	Hard Drive	SSD	Hard Drive
Operating System	Ubuntu	OS X	Scientific Linux
	14.04.1 LTS	10.10 (14A389)	2.6.18-164.10.1.el5
Java Version	1.8	1.8	1.8
Browser	Google Chrome	Safari	curl (command line)

### Connection validation

In order to evaluate the software, we took the approach of using a previously-published Histone Deacetylase (HDAC) inhibitor signature, as used in [6], for comparison. We present this signature to the following three configurations of software and data -

1. CMap
2. sscMap with CMap data set.
3. QUADrATiC with FDA-Approved Drug LINCS subset.

The concordance between 1 & 2 allows the connections derived from the new data set to be qualitatively evaluated, while that between 2 & 3 allows an evaluation of the new algorithm (as outlined previously) and its performance to be made.

**Fig. 6:**
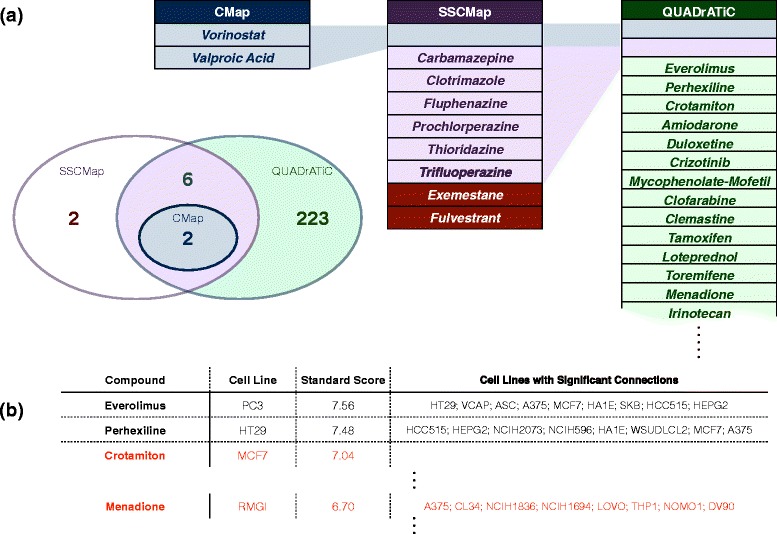
QUADrATiC Validation against HDAC Signature. **a** Venn diagram showing FDA-approved positive connections for existing sscMap software (*purple*) and QUADrATiC (*green*) and **b** Top QUADrATiC-only connections for treatment sets defined by drug and cell line. Results not found in the equivalent LINCS query are highlighted in red

QUADrATiC returns a list of 447 significant positive connections to the HDAC signature, which comprises 231 unique compounds over a number of different cell lines. Figure 6(a) summarizes the results and compares to the published results from sscMap [6] (Full details are available in Additional file 3). Note that the results have been filtered to extract only the FDA-approved positive connections from both CMap and sscMap. Of the ten connections identified by sscMap, eight are also identified by QUADrATiC. The two compounds not identified, Exemestane and Fulvestrant are calculated by QUADrATiC to have positive connections to the signature, but the estimated *p*-value does not allow these connections to be identified as significant. In addition to these connections, QUADrATiC identifies 223 additional compounds with significant positive connection scores. Figure 6(b) shows the top unique compounds with the strongest connections in this set of 223. As further validation, the HDAC signature was presented to the LINCS server through its query tool and the results from that query obtained (see the Additional file 4 for the full results). Of the ten best compounds identified by QUADrATiC, six are also identified as having strong positive connections in LINCS. Two of these remaining compounds are highlighted in red in Fig. 6(b). Of particular interest is Menadione, or Vitamin K3. Interestingly, a review of the literature produces evidence that Vitamin K3 acts to inhibit HDAC6, [24], suggesting that this connection has been correctly identified by QUADrATiC.

### Application - drug connections for a primary myelofibrosis signature

**Fig. 7:**
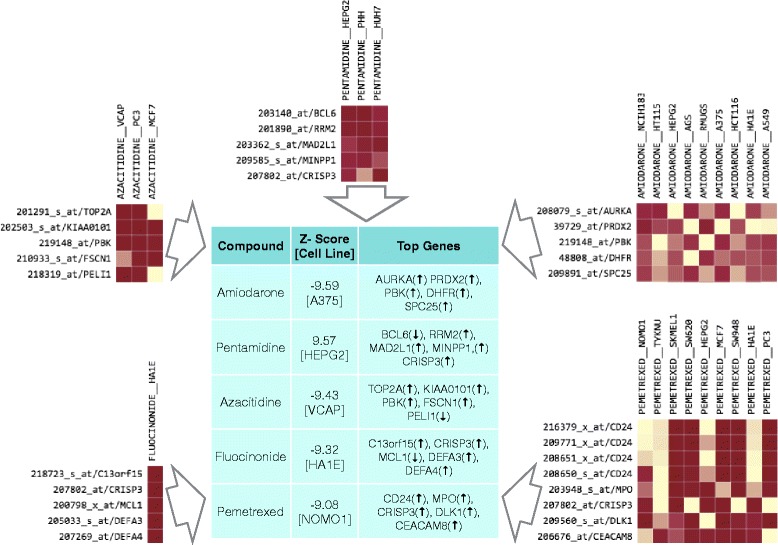
Top Five Negative Connections for PMF Signature. The table shows the detail of the top five negative connections found for the PMF signature discussed here. The peripheral figures show the normalized Contribution Fraction heat map for all significant negative connections to treatment sets with for each drug, as determined by QUADrATiC

In order to demonstrate application of QUADrATiC, it was applied to novel discovery for Myeloproliferative Neoplasms (MPN). In MPN, clonal proliferation of haematopoietic stem cells is often linked with an underlying genetic aberration [25]. In recent years a significant link has been uncovered between these conditions and a range of genes, most notably JAK2 and CALR. As a result, there is an emerging consensus that rather than being separate conditions, the three main types of BCR-ABl negative MPN, Essential Thrombocythaemia (ET), Polycythaemia Vera (PV) and Primary Myelofibrosis (PMF) are stages on a continuum of disease progression [26]. Around 10 % of PV cases (and 5 % in ET) are thought to transform to the more disabling MPN-associated Myelofibrosis (MF) [27], so any drugs which correlate to the reversal of cell states associated with this phenotype may profer a significant benefit to patients. Currently, much effort is being expended in developing JAK inhibitors (such as Ruxolitinib) as an alternative to first-line treatment options for MPN such as Hydroxyurea or Interferon alpha [28, 29].A publically-available dataset was identified, which was originally used to identify up-regulated genes in myelofibrosis [30]. This data series, GSE26049, was sourced from Gene Expression Omnibus (GEO) [31] and downloaded in normalized and background-corrected form. This series consists of whole blood expression data from 91 subjects (19 with Essential Thrombocythemia, 41 with Polycythemia Vera, 9 with Primary Myelofibrosis, 1 with Unclassified Myeloproliferative Disorder, and 21 controls). Using this data, two groups were defined: the group of subjects diagnosed with PV, and the group of subjects with PMF. The expression data for these two groups was extracted and analyzed using the limma package [32] in R to identify the significantly up and down-regulated genes. The R statistical package is freely downloadable software [33] containing many peer reviewed packages that can be used in different biological statistical analyses. The signature was created using those probes with fold change greater than two, and adjusted for a false discovery rate of 0.05, using the Benjamini-Hochberg criteria. The signature and the full set of genes and Affymetrix probe IDs is available in the Additional file 5. This signature was presented to the QUADrATiC software, and the list of significant negative connections (i.e. those which are seen to reverse the phenotype) retrieved. The top connections in the list are analysed for existing or previous use in treatment of myelofibrosis, as detailed in two recent publications [28, 34]. Presenting the signature for myelofibrosis discussed above to QUADrATiC, and calculating connections for treatments sets aggregated by Drug resulted in 899 signficant negative connections to drug/cell line treatment sets. Table 2 shows the connections found (if any) to the combined lists of FDA-approved drugs identified as being in current use for the treatment of myelofibrosis (from [28, 34]). Apart from the compounds identified in Table 2, there are 385 other compounds with significant negative connections to the myelofibrosis signature (of which 188 have negative connections in two or more cell lines). By comparison, analyzing the signature using sscMap results in 412 connections for compounds in more than one cell line, but the majority of those connections are not FDA-approved drugs. Figure 7 shows the top five compounds with negative connections to the PMF signature, along with the five genes having the highest median value of $CF^{*}_{k}$, across all significant negative treatment sets for the same drug. Of these 5 drugs, 4 are also identified by sscMap, but Pemtrexed is not, as there are no reference profiles for that compound in the original Cmap data set, from which sscMap derives its profiles.

1. **Amiodarone** is typically used to treat cardiac arrhythmias. Its use to treat leukemia has been investigated with some success in mice [35].

**Table 2:** Existing PMF treatments (from [28, 34]) in significant connection set

Drug	Comments	Cell lines
Anagrelide	Significant negative connections found	ASC, NPC
Azacitidine	Significant negative connections found	VCAP, PC3, MCF7
Busulfan	No significant negative connections found	
Cytarabine	No significant negative connections found	HA1E, PC3, A549, MCF7, VCAP
	(indicative negative connections found)	
Danazol	Significant negative connections found	PHH, ASC
Decitabine	No significant negative connections found	
Epoetin-alpha	No data in LINCS set	
Everolimus	Significant negative connections found	A549, ASC, HT29
Hydroxyurea	No data in LINCS set	
Interferon alpha	No data in LINCS set	
Lenalidomide	Significant negative connections found	SNUC4, A375, COV644
Melphalan	No significant negative connections found	HA1E
	(indicative negative connection found)	
Mercaptopurine	Significant negative connection found	MCF7
Methylprednisolone	Significant negative connection found	A549
Pomalidomide	No data in LINCS set	
Prednisone	No significant negative connections found	
Ruxolitinib	Significant negative connection found	HEPG2
Thalidomide	Significant negative connections found	TYKNU, PHH, CL34, HCC515
Thioguanine	No data in LINCS set
  Amiodarone has been shown to have hematological effects, and interactions with warfarin [36], so this may provide an avenue for its investigation.
2. **Pentamidine** is an antiprotozoal drug, used to treat fungal infections and pneumonia. It has been investigated for potential anticancer activity, including its use in treatment of chronic myelogenous leukemia [37].
  One possible mechanism of action of pentamidine could lie in its inhibitory action on S100B [38], which in turn interacts with p53 [39]. There is some evidence that p53 is linked to progression of MPN [40].
3. **Azacitidine** has, as mentioned earlier been used to treat myelofibrosis in a clinical context.
4. **Pemetrexed** is an antifolate drug which is used in the treatment of non-small cell lung cancer [41].
  Pemetrexed has been shown to be an inhibitor of DHFR [42]; another DHFR inhibitor, methotrexate, has recently been shown to inhibit the JAK/STAT pathway, which is a key biological pathway in MPN [43].
5. **Fluocinonide** is a glucocorticoid used as an anti-inflammatory in the treatment of eczema and other skin disorders.
  Prednisone, another glucocorticoid, is currently used in the treatment of PMF [34].

Using the Contribution Fraction feature of QUADrATiC allows these connections to be analysed further to narrow down the list of genes involved in returning this drug and also allows biology-based researchers to identify and suggest possible mechanisms of action for the drugs in a biological context. For example, taking the five drugs highlighted above, it is possible to extract that subset of the signature with positive CF. The biological implications and the overall “ontotype” of these signature subsets were further analyzed through the use of QIAGEN Ingenuity®; Pathway Analysis (IPA®;, QIAGEN Redwood City, www.qiagen.com/ingenuity). The detail of the results from this analysis can be viewed in the Additional file 6, which shows the results of the Canonical Pathway, Diseases & Functions and Networks analyses (specifically, given the nature of the disease, the networks corresponding to haematological system development and function). Each of the drugs appears to impact transcription of genes associated with aspects of Haematological System Development and Function, and NF *κ*B-mediated Inflammatory Response in particular, suggesting that they may act on these mechanisms in providing potential therapeutic effect in Myelofibrosis. Using QUADrATiC allows the analysis of 83,939 references (aggregated into 10,174 treatment sets) from the LINCS data set to produce a list of 899 significant negative connections, covering 405 distinct drugs. This list contains many of the widely-used existing clinical treatments for PMF, and suggests a multitude of others which offer potential for further study, and possibly therapeutic benefit to patients. Since QUADrATiC works with the subset of LINCS corresponding to the list of currently FDA-approved drugs, the drugs corresponding to these connections are likely to be more-widely studied, with mechanisms of action which are better understood.

## Discussion and conclusions

Taking a software engineering approach to the design and development of gene expression connectivity mapping, in particular making use of the concurrent actor paradigm, allows the performance of a previously-sequential algorithm to be scaled to handle much larger data sets available from LINCS. The order of improvement of performance is similar to that shown for previous parallel implementation using GPGPU technology [11]. However, QUADrATiC’s performance is achieved on widely-available standard computing hardware (including a laptop) and does not require additional co-processor cards, or the use of esoteric, difficult-to-use programming models such as CUDA. This allows easier development and debugging, using standard tools and languages such as Java. In addition, through the use of Java interfaces, the algorithm used by the Scorers may be updated. At present, this requires recompilation, but in the future it is possible that the capability to create and dynamically update the scoring algorithm will be available to the end user, opening up the possibility of other innovative analyses of the data provided. Indeed, QUADrATiC is one of the first applications of the Akka framework within the genomics/bioinformatics domain and shows how it may be used to take advantage of modern CPU-based parallelism to efficiently process larger quantities of data than is feasible with previous approaches. Akka, and the actor paradigm, is of course only one such approach to processing larger quantities of data, and GPGPU technology as in [11] could also provide an alternative approach to implementing highly parallel algorithms. However, current GPGPU technology tends only to have limited on-board memory capabilities, which tends not to lend itself to larger amounts of reference data such as LINCS. Given the current implementation, QUADrATiC will scale with processors and memory in a single server, so in its current form, the applicable data set will be limited by these. However, within the Akka framework, there is the capability to distribute so-called ’remote actors’ across multiple servers (either ’bare metal’ servers or virtual machine instances in the cloud) and thus act in a similar manner to the currently-popular Hadoop distributed processing platform. The implementation of QUADrATiC as a web server application, exposing a JSON interface over HTTP allows the use of modern GUI development frameworks to provide a simple, reactive user interface, and opens the way to deployment on larger server systems as a shared resource. In addition, the availability of a programmatic web service interface brings into consideration the possibility of using connectivity mapping within the larger Integromics frameworks becoming available [44]. The development of this software has integrated the requirements of the biology end user at each stage by providing easy to understand quantative and qualitatiove outputs, which all a rapid and relevant interpretation of the data. Importantly, the built in features allow the laboratory-based validation of the mechanism of action by providing a short list of the most relevant biomarkers which should be used when validating these results in vitro or in vivo. In addition, as we have clearly demonstrated through the use of ontotype analysis, this data is in a ready to use format for directly exporting into downstream tools.

We have shown that QUADrATiC compares well to the existing connectivity map implementations, based on the CMap data set, and also identifies additional connections over and above those provided as standard by the LINCS Query Tool. Being based on a specific subset of the LINCS data (that of FDA-approved drugs), it serves the purpose that any connections found are likely to be more easily obtained for study and allowing for the progress of any successful therapeutics into clinical use. Defining a novel signature for Primary Myelofibrosis from a high quality, publicly-available data set, we were able to identify a large number of negative (i.e. potentially therapeutic) connections within the QUADrATiC reference data set. Confidence in this list of connections is greatly increased by the presence of a large number of existing PMF treatments. However, QUADrATiC returns a much larger list of candidates, which can be studied further in vitro. Indeed, the large number of results returned for connections, as a result of the vastly larger reference data set will likely require development of strategies for filtering results (based on integration with chemical data, mining related publications and/or cell line/mutation details) to give a manageable list. As a start, we have shown that the new Contribution Fraction feature of QUADrATiC provides an output which is easy to interpret for end users with limited computation knowledge a visual representation of the underlying biology and mechanisms of action of candidate therapeutics. This feature provides the wet lab biologist with important information for selecting the appropriate control biomarkers to confirm the success of drug treatment which can be analysed further using downstream tools when testing in the laboratory, which is, after all, the final arbiter of the utility of a drug. In conclusion, QUADrATiC has been shown to provide an improvement over existing connectivity map software, in terms of scope (based on the LINCS data set), applicability (using FDA-approved compounds), usability and speed. It offers potential to biological researchers to analyze transcriptional data and generate potential therapeutics for focussed study in the lab.

## Ethics approval and consent to participate

Not applicable.

## Consent for publication

Not applicable.

## Availability of data and materials

**Project name**: QUADrATiC**Project home page**: http://go.qub.ac.uk/QUADrATiC**Operating systems**: Platform independent**Programming language**: Java, HTML, and Javascript**Other requirements**: Java 1.8 or higher**License**: Creative Commons license by-nc 4.0**Any restrictions to use by non-academics**: For commercial use, please contact the authors.The datasets supporting the conclusions of this article are included within the article and its additional files.

## Additional files

Additional file 1 Brief user manual for installing and using QUADrATiC. (PDF 1024 kb)

Additional file 2 The specification of the basic REST API supported by QUADrATiC. (XLSX 9.76 kb)

Additional file 3 Spreadsheet file with the full list of significant positive connections from QUADrATiC for the HDAC signature used to validate the software. (XLSX 84.0 kb)

Additional file 4 Spreadsheet file with the results summary file as downloaded from LINCS, for the HDAC signature used to validate the software. (CSV 4259.84 kb)

Additional file 5 Spreadsheet file with full details of, a) differential expression analysis results for GSE26049, b) list of probes used in the signature, and c) full results from QUADrATiC. (XLSX 5457.92 kb)

Additional file 6 The output from Qiagen IPA for the contributing genes for the top 5 drug connections. (PDF 2068.48 kb)

## Abbreviations

API: application program interface
CF: contribution fraction
ET: essential thrombocythaemia
FPGA: field-programmable gate arrays
GPGPU: general purpose graphics processing units
GUI: graphical user interface
HDD: hard disk drive
HTTP: hypertext transfer protocol
JVM: java virtual machine
LINCS: library of integrated cellular signatures
MPN: myeloproliferative neoplasms
M2M: machine-machine
NGS: next generation sequencing
PMF: primary myelofibrosis
PV: polycythaemia vera
QUADrATiC: QUB accelerated drug and transcriptomic connectivity
SSD: solid-state drive
URI: uniform resource identifier

## References

[1] Lamb J, Crawford ED, Peck D, Modell JW, Blat IC, Wrobel MJ, Lerner J, Brunet JP, Subramanian A, Ross KN, Reich M, Hieronymus H, Wei G, Armstrong SA, Haggarty SJ, Clemons PA, Wei R, Carr SA, Lander ES, Golub TR (2006). The connectivity map: using gene-expression signatures to connect small molecules, genes, and disease. Science.

[2] Qu XA, Rajpal DK (2012). Applications of connectivity map in drug discovery and development. Drug Discov Today.

[3] Iorio F, Rittman T, Ge H, Menden M, Saez-Rodriguez J (2013). Transcriptional data: a new gateway to drug repositioning?. Drug Discov Today.

[4] McArt DG, Dunne PD, Blayney JK, Salto-Tellez M, Schaeybroeck SV, Hamilton PW, Zhang SD (2013). Connectivity mapping for candidate therapeutics identification using next generation sequencing rna-seq data. PloS ONE.

[5] The Connectivity Map 02. http://www.broadinstitute.org/cmap. Accessed 15 January 2016.

[6] Zhang SD, Gant TW (2008). A simple and robust method for connecting small-molecule drugs using gene-expression signatures. BMC Bioinformatics.

[7] Zhang SD, Gant TW (2009). sscmap: an extensible java application for connecting small-molecule drugs using gene-expression signatures. BMC Bioinformatics.

[8] Ramsey JM, Kettyle LMJ, Sharpe DJ, Mulgrew NM, Dickson GJ, Bijl JJ, Austin P, Mayotte N, Cellot S, Lappin TRJ, Zhang SD, Mills KI, Krosl J, Sauvageau G, Thompson A (2013). Entinostat prevents leukemia maintenance in a collaborating oncogene-dependent model of cytogenetically normal acute myeloid leukemia. Stem Cells.

[9] Smalley JL, Gant TW, Zhang SD (2010). Application of connectivity mapping in predictive toxicology based on gene-expression similarity. Toxicology.

[10] Wen Q, O’Reilly P, Dunne PD, Lawler M, Schaeybroeck SV, Salto-Tellez M, Hamilton P, Zhang SD (2015). Connectivity mapping using a combined gene signature from multiple colorectal cancer datasets identified candidate drugs including existing chemotherapies. BMC Syst Biol.

[11] McArt DG, Bankhead P, Dunne PD, Salto-Tellez M, Hamilton P, Zhang SD (2013). cudamap: a gpu accelerated program for gene expression connectivity mapping. BMC Bioinformatics.

[12] Buchty R, Heuveline V, Karl W, Weiss JP (2012). A survey on hardware-aware and heterogeneous computing on multicore processors and accelerators. Concurr Comput Pract Experience.

[13] Hasselbring W (2000). Programming languages and systems for prototyping concurrent applications. ACM Comput Surv.

[14] Haller P, Odersky M (2009). Scala actors: unifying thread-based and event-based programming. Theor Comput Sci.

[15] Lee EA (2006). The problem with threads. Computer.

[16] Build powerful concurrent and distributed applications more easily. http://akka.io/. Accessed 15 January 2016.

[17] Margaria T, Steffen B (2014). Leveraging Applications of Formal Methods, Verification and Validation. Specialized Techniques and Applications: 6th International Symposium, ISoLA 2014, Imperial, Corfu, Greece, October 8-11, 2014, Proceedings, Part II.

[18] Fuller JC, Martinez M, Henrich S, Stank A, Richter S, Wade RC (2015). Ligdig: a web server for querying ligand-protein interactions. Bioinformatics.

[19] Law V, Knox C, Djoumbou Y, Jewison T, Guo AC, Liu Y, Maciejewski A, Arndt D, Wilson M, Neveu V, Tang A, Gabriel G, Ly C, Adamjee S, Dame ZT, Han B, Zhou Y, Wishart DS (2014). Drugbank 4.0: shedding new light on drug metabolism. Nucleic Acids Res.

[20] MacGillivray HL (1986). Skewness and asymmetry: measures and orderings. Ann Stat.

[21] Balasubramanee V, Wimalasena C, Singh R, Pierce M (2013). Twitter bootstrap and angularjs: Frontend frameworks to expedite science gateway development. Cluster Computing (CLUSTER), 2013 IEEE International Conference On.

[22] Bostock M, Ogievetsky V, Heer J (2011). D(3): Data-driven documents. IEEE Trans Vis Comput Graph.

[23] Richardson L (2007). RESTful Web Services.

[24] Rahn JJ, Bestman JE, Josey BJ, Inks ES, Stackley KD, Rogers CE, Chou CJ, Chan SS (2014). Novel vitamin k analogs suppress seizures in zebrafish and mouse models of epilepsy. Neuroscience.

[25] Tefferi A, Pardanani A (2015). Myeloproliferative neoplasms: A contemporary review. JAMA Oncol.

[26] Tefferi A, Vainchenker W (2011). Myeloproliferative neoplasms: molecular pathophysiology, essential clinical understanding, and treatment strategies. J Clin Oncol Off J Am Soc Clin Oncol.

[27] Tefferi A (2014). Primary myelofibrosis: 2014 update on diagnosis, risk-stratification, and management. Am J Hematol.

[28] Harrison C, Kiladjian JJ, Al-Ali HK, Gisslinger H, Waltzman R, Stalbovskaya V, McQuitty M, Hunter DS, Levy R, Knoops L, Cervantes F, Vannucchi AM, Barbui T, Barosi G (2012). Jak inhibition with ruxolitinib versus best available therapy for myelofibrosis. N Engl J Med.

[29] Vannucchi AM, Kantarjian HM, Kiladjian JJ, Gotlib J, Cervantes F, Mesa RA, Sarlis NJ, Peng W, Sandor V, Gopalakrishna P, Hmissi A, Stalbovskaya V, Gupta V, Harrison C, Verstovsek S. A pooled analysis of overall survival in comfort-i and comfort-ii, 2 randomized phase 3 trials of ruxolitinib for the treatment of myelofibrosis. Haematologica. 2015; 100:1139–1145. 10.3324/haematol.2014.119545 PMC4800694 26069290

[30] Skov V, Larsen TS, Thomassen M, Riley CH, Jensen MK, Bjerrum OW, Kruse TA, Hasselbalch HC (2011). Whole-blood transcriptional profiling of interferon-inducible genes identifies highly upregulated ifi27 in primary myelofibrosis. Eur J Haematol.

[31] Gene Expression Omnibus (GEO) Data Series GSE26049. http://www.ncbi.nlm.nih.gov/geo/query/acc.cgi?acc=GSE26049. Accessed 15 November 2015.

[32] Ritchie ME, Phipson B, Wu D, Hu Y, Law CW, Shi W, Smyth GK (2015). limma powers differential expression analyses for rna-sequencing and microarray studies. Nucleic Acids Res.

[33] The R Project for Statistical Computing. http://www.r-project.org/. Accessed 15 January 2016.

[34] Mesa RA (2013). The evolving treatment paradigm in myelofibrosis. Leukemia & Lymphoma.

[35] Papageorgiou AD, Dalezis P, Mourelatos C, Lioutas K, Sahpazidou D, Geromichalou E, Geromichalos G, Lialiaris T, Athanasiadou P, Athanasiadis P (2010). Preclinical evaluation of amiodarone for the treatment of murine leukemia p388. In vivo and in vitro investigation. J BUON Off J Balkan Union Oncol.

[36] Flaker G, Lopes RD, Hylek E, Wojdyla DM, Thomas L, Al-Khatib SM, Sullivan RM, Hohnloser SH, Garcia D, Hanna M, Amerena J, Harjola VP, Dorian P, Avezum A, Keltai M, Wallentin L, Granger CB (2014). Amiodarone, anticoagulation, and clinical events in patients with atrial fibrillation: insights from the aristotle trial. J Am Coll Cardiol.

[37] Qiu G, Jiang J, Liu XS (2012). Pentamidine sensitizes chronic myelogenous leukemia k562 cells to trail-induced apoptosis. Leuk Res.

[38] Smith J, Stewart BJ, Glaysher S, Peregrin K, Knight LA, Weber DJ, Cree IA (2010). The effect of pentamidine on melanoma ex vivo. Anti-Cancer Drugs.

[39] Gieldon A, Mori M, Conte RD (2007). Theoretical study on binding of s100b protein. J Mol Model.

[40] Du Y, Chen Y, Ho W, Zhao ZJ (2013). The role of p53 in jak2v617f-induced myeloproliferative neoplasms. Blood.

[41] Hanna N, Shepherd FA, Fossella FV, Pereira JR, Marinis FD, von Pawel J, Gatzemeier U, Tsao TC, Pless M, Muller T, Lim HL, Desch C, Szondy K, Gervais R, Manegold C, Paul S, Paoletti P, Einhorn L, Jr PAB, Shaharyar (2004). Randomized phase iii trial of pemetrexed versus docetaxel in patients with non-small-cell lung cancer previously treated with chemotherapy. J Clin Oncol Off J Am Soc Clin Oncol.

[42] Gibbs D, Jackman A (2005). Pemetrexed disodium. Nat Rev Drug Discov.

[43] Thomas S, Fisher K, Snowden J, Danson S, Brown S, Zeidler M (2015). Effect of methotrexate on jak/stat pathway activation in myeloproliferative neoplasms. The Lancet.

[44] McArt DG, Blayney JK, Boyle DP, Irwin GW, Moran M, Hutchinson RA, Bankhead P, Kieran D, Wang Y, Dunne PD, Kennedy RD, Mullan PB, Harkin DP, Catherwood MA, James JA, Salto-Tellez M, Hamilton PW (2015). Pican: an integromics framework for dynamic cancer biomarker discovery. Mol Oncol.

