# Supplementary material for: QUADrATiC: scalable gene expression connectivity mapping for repurposing FDA-approved therapeutics
Source: BMC Bioinformatics. 2016 May 4;17:198. doi: 10.1186/s12859-016-1062-1 (PMC4855472; doi:10.1186/s12859-016-1062-1)
Supplement: Additional file 1 — Brief user manual for installing and using QUADrATiC. (PDF 1024 kb) [file 12859_2016_1062_MOESM1_ESM.pdf]

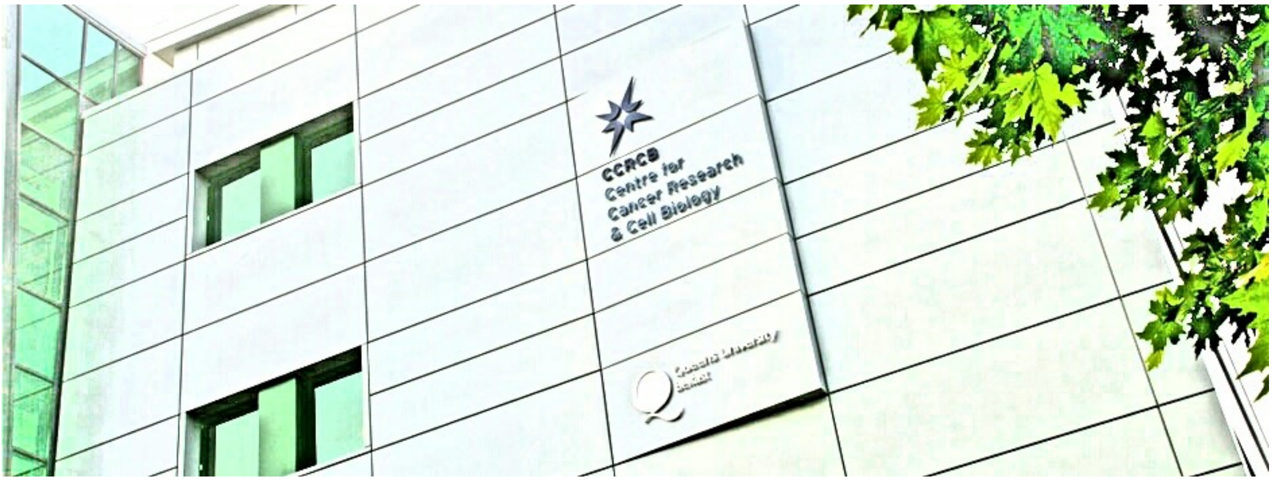

QUADrATiC User Manual

Paul O'Reilly
CCRCB
Queen's University Belfast
8 October 2015

INSTALLING AND STARTING THE SOFTWARE

User Goals

You have downloaded QUADrATiC and wish to run the program on a computer.

Preconditions

Linux/Mac/Windows machine with > 4GB RAM and multicore processor (ideally with hyperthreading enabled).

Java 1.7 and above

15GB spare disk space

Steps

1. Unzip the QUADrATiC.zip file in a suitable location on your hard drive.

   [NOTE: on Windows there are limitations of file name & path length, so it is suggested that you extract in your root directory (typically C:\)]

2. Open a command line window (the following instructions are for linux – those for Windows are very similar).

3. Change to QUADrATiC root folder, and type ls (Linux/Mac)/ dir (Windows) to check the required files are OK (see below)

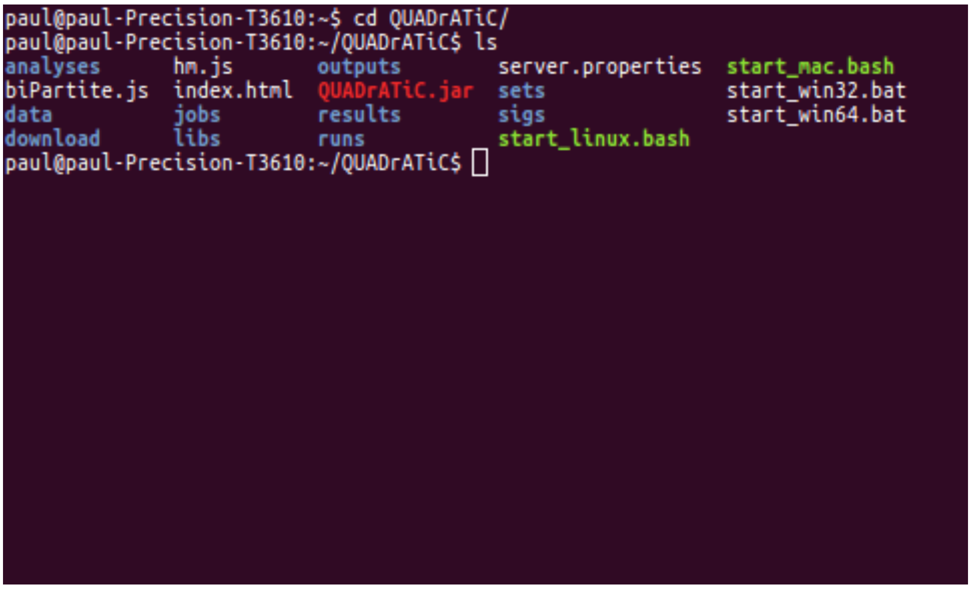

4. Use the relevant start file (start_linux.bash for 64-bit linux, start_mac.bash for 64-bit mac, start_win32.bat for 32-bit windows, and start_win64.bat for 64-bit windows) to start the program. The default setting should be OK, but if there are any problems on startup (or indeed afterwards), it is possible to hand-set certain properties in the "server.properties" file.

```
paul@paul-Precision-T3610:~/QUADrATiC$ ./start_linux.bash
2015-10-08 09:47:33.182:INFO::main: Logging initialized @1186ms
Current relative path is: /home/paul/QUADrATiC
###############
SERVER SETTINGS
###############

-----------------------
Directory Configuration
-----------------------
ROOT_DIR: ./
RESULT_DIR: ./results/
JOB_DIR: ./jobs/
ANALYSIS_DIR: ./analyses/
SET_DIR: ./sets/
SIG_DIR: ./sigs/
DATA_DIR: ./data/
OUTPUT_DIR: ./outputs/

------------------
HTTP Configuration
------------------
HTTP_LISTEN_PORT: 8090
NUM_HTTP_THREADS: 6

--------------------
Worker Configuration
--------------------
NUM_SCORERS: 15
2015-10-08 09:47:33.359:INFO:oejs.Server:main: jetty-9.2.9.v20150224
2015-10-08 09:47:33.441:INFO:oejs.ServerConnector:main: Started ServerConnector@
27082746{HTTP/1.1}{0.0.0.0:8090}
2015-10-08 09:47:33.442:INFO:oejs.Server:main: Started @1476ms
```

DEFINING THE SIGNATURE

User Goals

You want to create a new signature for a connectivity map analysis.

Preconditions

QUADrATiC is installed and running on an accessible computer.

Steps

1. In a browser (i've tested most in Safari & Chrome, but any modern browser should work), open the URL -

   http://localhost:8090

   This should load the page shown here.

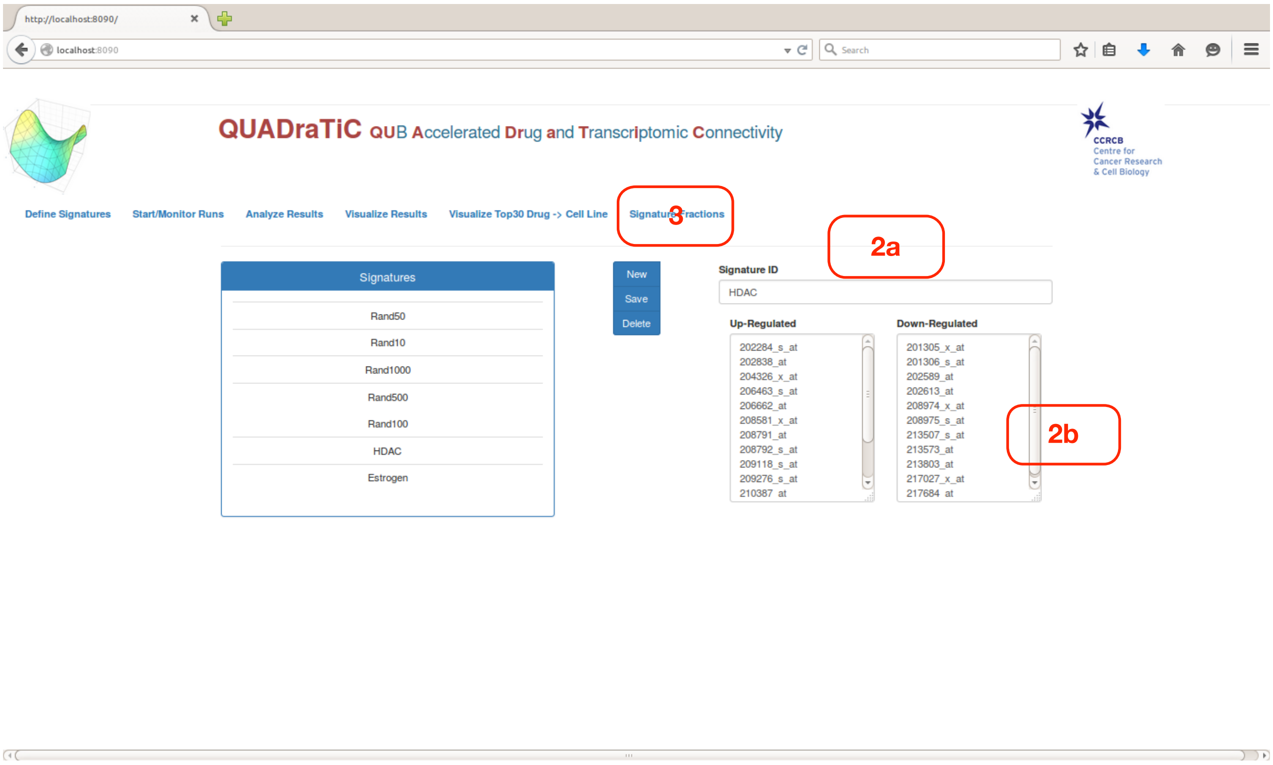

[The web interface defaults to the ‘Define Signatures’ tab.]

2. Enter (a) a signature name (in this case, “HDAC”), and (b) paste the lists of up-regulated and down-regulated probes into the requisite sections.

3. Press 'Save' and the signature should be created.

[Alternatively you can pick an existing signature from the ‘Signatures’ list on the left-hand side of the screen]

RUNNING A CONNECTIVITY MAP ANALYSIS

User Goals

You want to find the connections for a gene signature.

Preconditions

The gene signature you want to use has been created in QUADrATiC.

Steps

1. Navigate to the ‘Start/Monitor Runs’ tab (below)

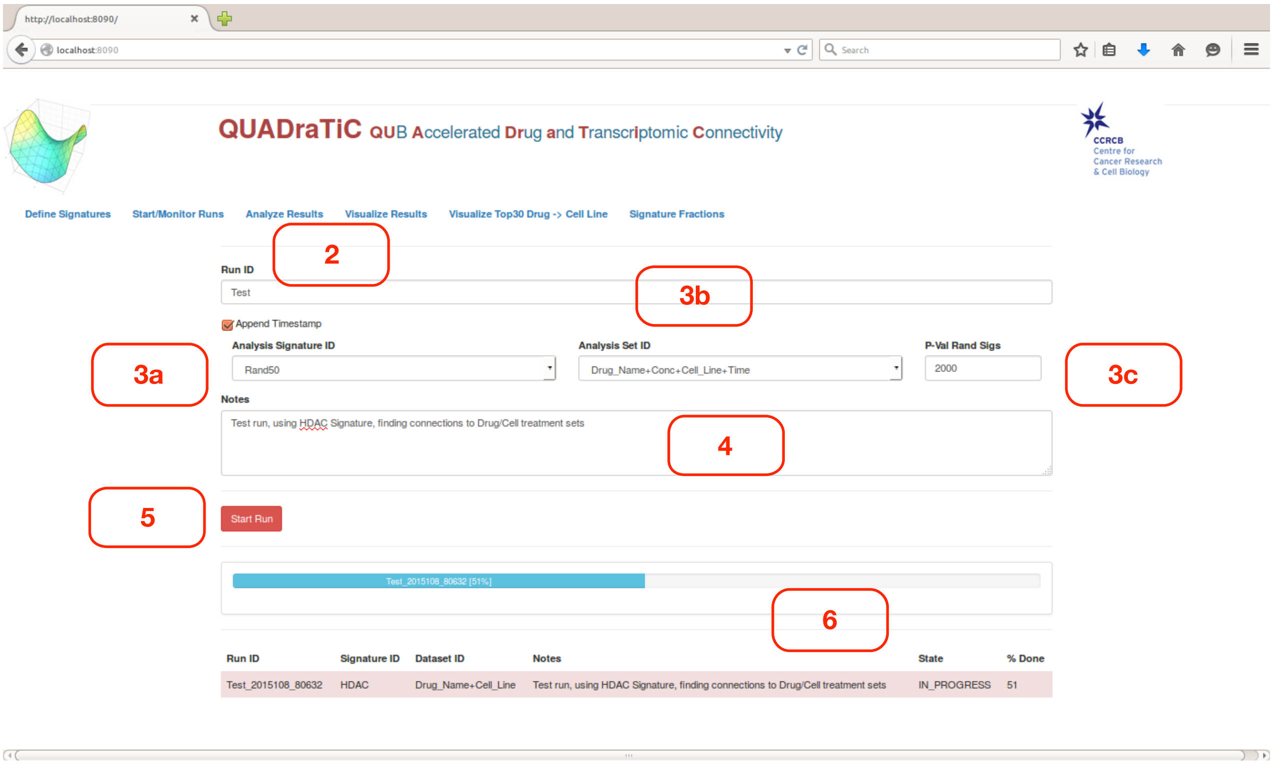

2. Enter a Run Identifier in the relevant field (if the ‘Append Timestamp’ box is checked, QUADrATiC will append a timestamp string to this ID to ensure uniqueness).

3. Choose (a) the signature, (b) the Reference Sets to use (Drug/Drug+Cell Line, etc.) and (c) the number of random signatures to use for p-value estimation (2000 is generally a good choice).

4. Add some notes if you want.

5. Press the Start button.

6. The analysis should start and the progress will be indicated in the lower panel of the screen.

ANALYZING THE RESULTS

User Goals

Having run an analysis you want to see some results.

Preconditions

The analysis you're interested in has been run and completed OK.

Steps

1. Navigate to the 'Analyze Results' tab (below).

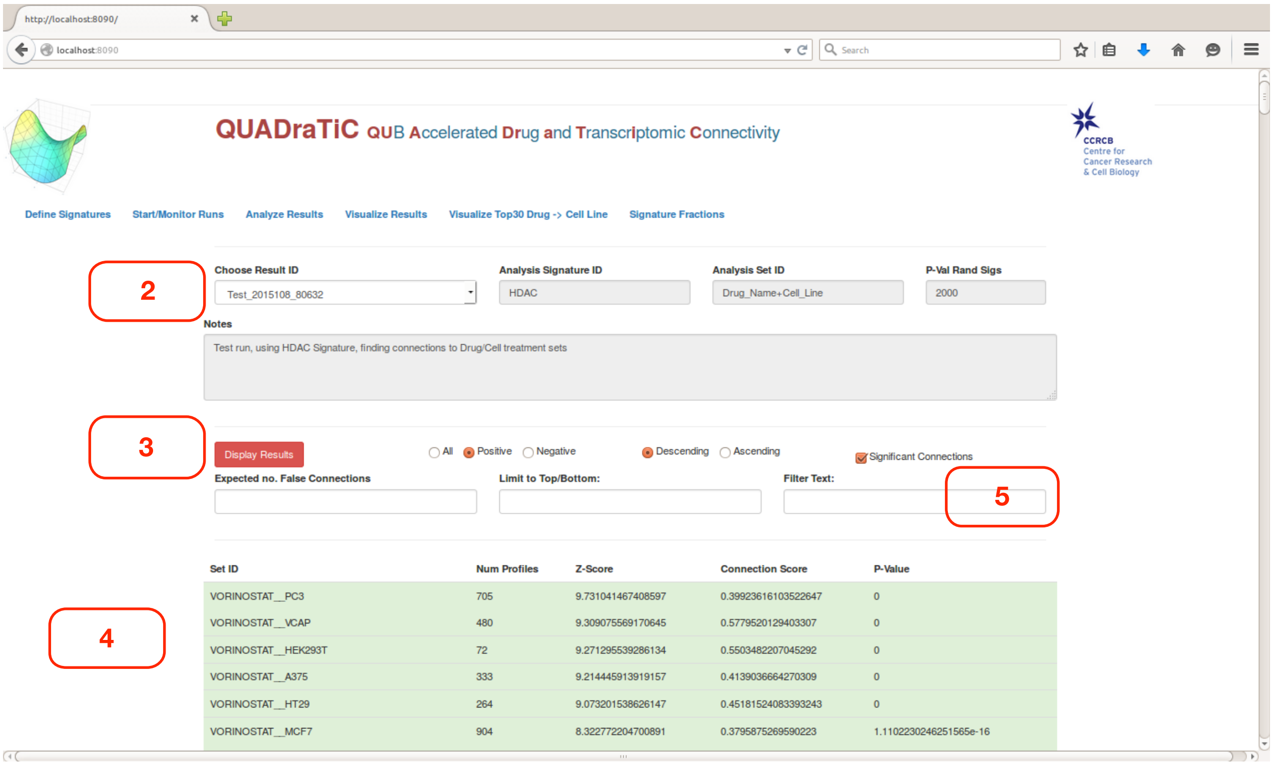

2. Select the result set you want to see the detail for.
3. Click 'Display Results'
4. The table of (by default) significant connections will be shown. You can scroll down through this.
5. You may sort (by z-score only) or apply a variety of filters to this view of the data.

PRODUCING A BUBBLE PLOT VISUALIZATION

User Goals

Having run an analysis you want to see a Bubble Plot of your results.

Preconditions

The analysis you're interested in has been run and completed OK.

Steps

1. Navigate to the 'Visualize Results' tab (below)

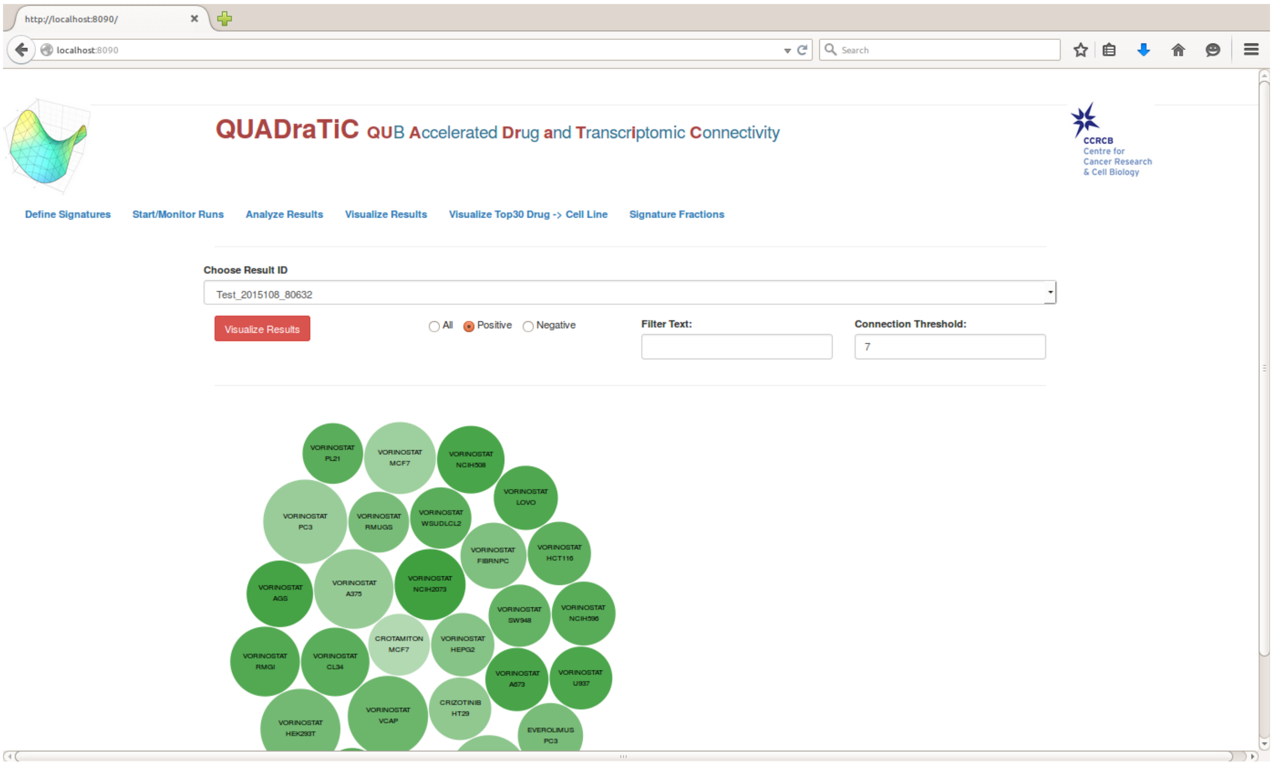

2. Load the result set as before and use the check boxes and filters to view a subset of the results in Bubble Plot form.

VISUALIZING THE TOP 30 CONNECTIONS

User Goals

Having run an analysis for the drug/cell line reference sets, you want to see a visualisation of the top positive or negative connections and their links to drugs and/or cell lines.

Preconditions

The analysis you're interested in has been carried out for the 'Drug+Cell Line' data set, run and completed OK.

Steps

1. Navigate to the 'Visualize Top30 Drug -> Cell Line' tab (below)

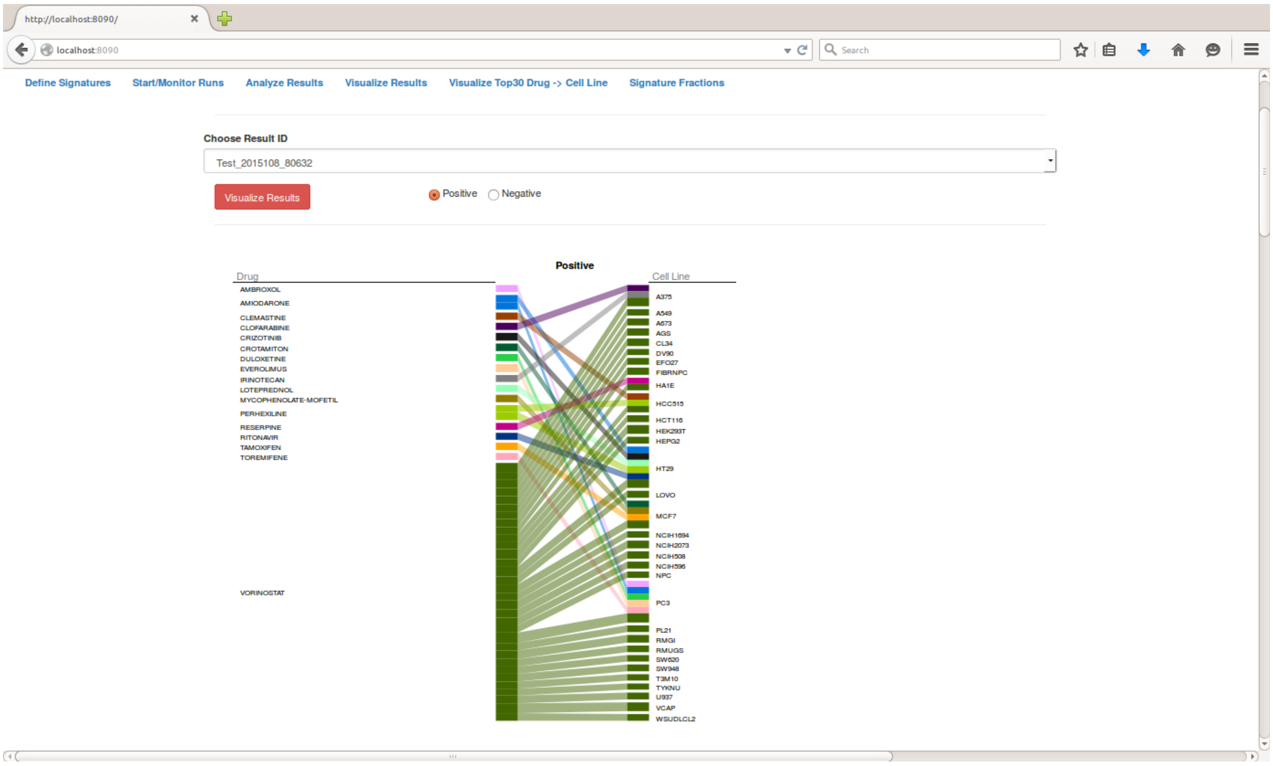

2. Load the results as before, choosing either the positive or negative connection set.

3. Hovering over a drug (LHS) or Cell Line (RHS) will accentuate the links for that entity (below)

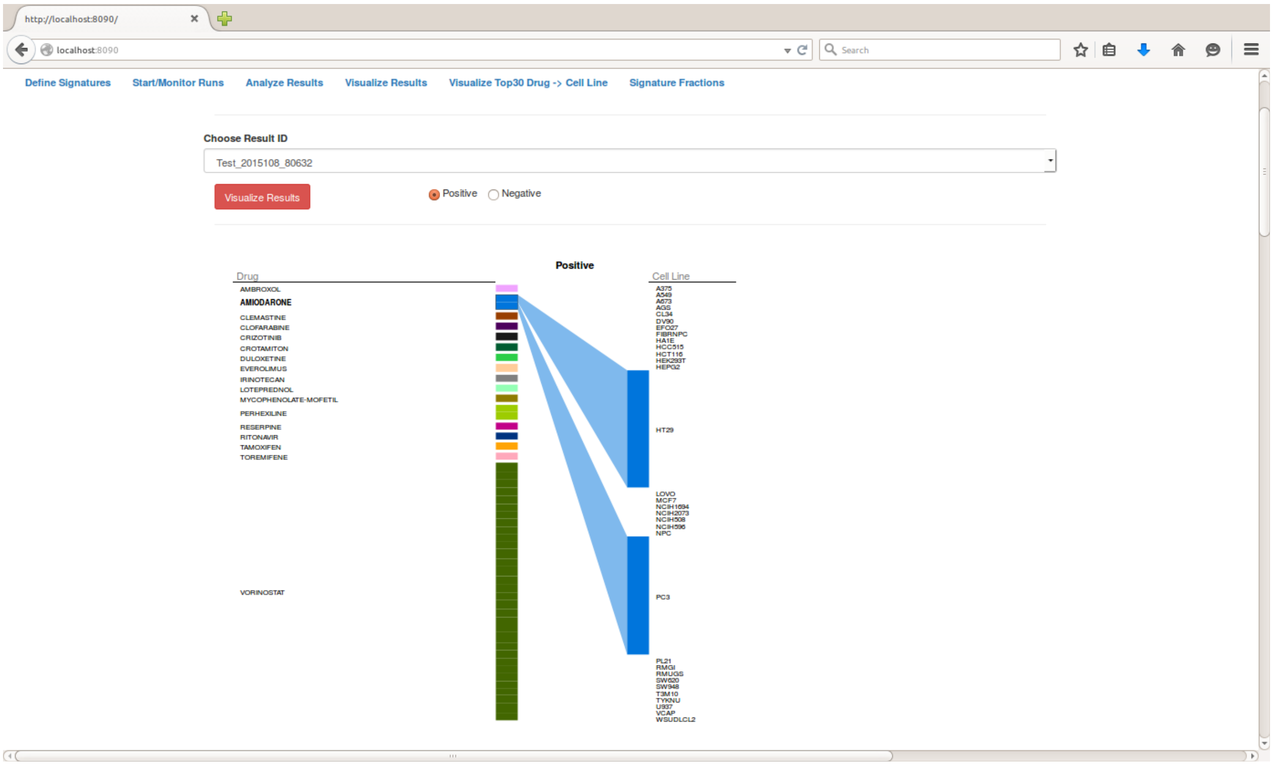
http://localhost:8090/
localhost:8090
Search
Define Signatures
Start/Monitor Runs
Analyze Results
Visualize Results
Visualize Top30 Drug -> Cell Line
Signature Fractions
Choose Result ID
Test_2015108_80632
Visualize Results
Positive
Negative
Positive
Drug
AMBROXOL
AMIODARONE
CLEMASTINE
CLOFARABINE
CRIZOTINIB
CROTAMITON
DULOXETINE
EVEROLIMUS
IRINOTECAN
LOTEPREDNOL
MYCOPHENOLATE-MOFETIL
PERHEXILINE
RESERPINE
RITONAVIR
TAMOXIFEN
TOREMIFENE
VORINOSTAT
Cell Line
A375
A549
A673
AGS
CL34
DV90
EFO27
FIBRNPC
HA1E
HCC515
HCT116
HEK293T
HEPG2
HT29
LOVO
MCF7
NCIH1694
NCIH2073
NCIH508
NCIH596
NPC
PC3
PL21
RMGI
RMUGS
SW620
SW948
T3M10
TYKNU
U937
VCAP
WSUDLCL2

VISUALIZING THE CONTRIBUTION FRACTIONS

User Goals

Having run an analysis for the drug/cell line reference sets, you want to see a visualisation of the contribution fractions of the top positive or negative connections.

Preconditions

The analysis you're interested in has been run and completed OK.

Steps

1. Navigate to the 'Signature Fractions' tab (below)

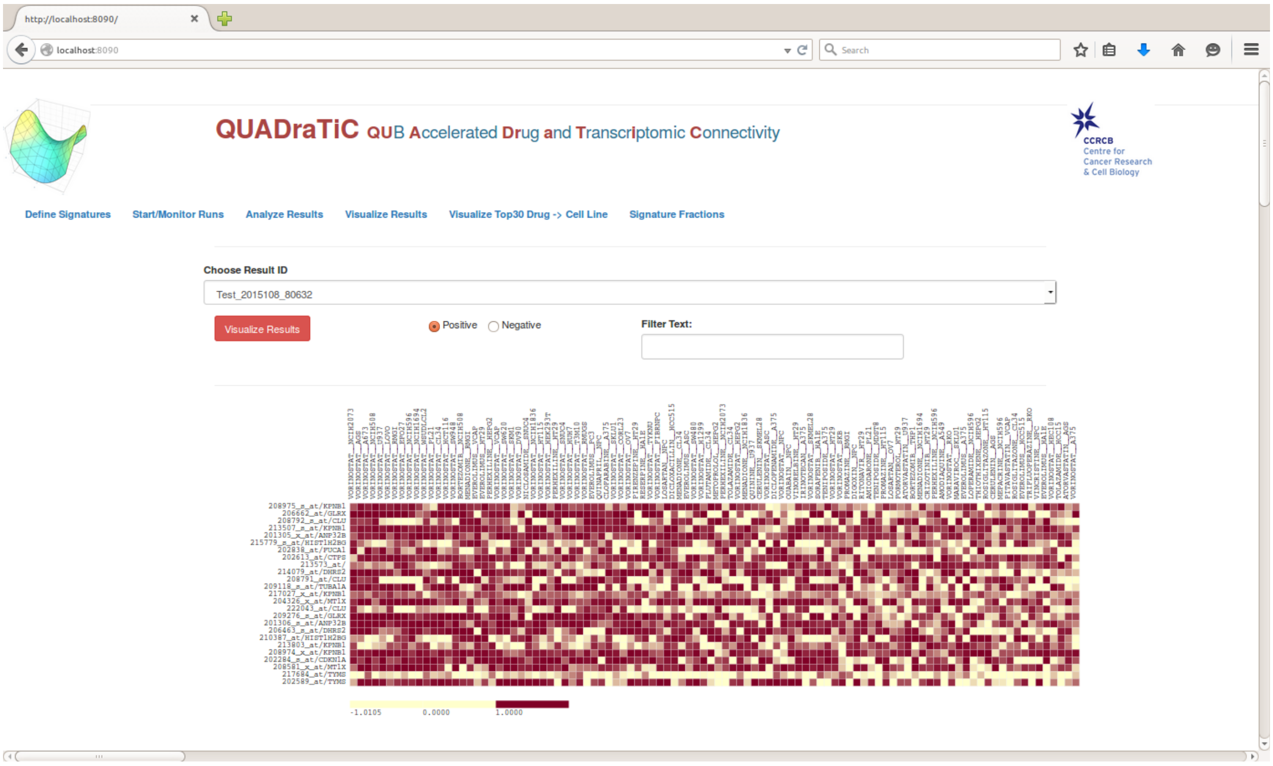

2. Load the results as before, choosing either the positive or negative connection set.

3. The heat map shows the contribution fractions (normalized) for the top 50 connections (matching the filter text if applicable). Hovering over a cell on the heat map gives the details of the CF.

4. Clicking the row header (to the left of the heat map) reorders the data by median CF of the entire row. This is not really useful for the broad set of results, but if a filter is applied to only show a particular drug or drug family, it can be useful to see which probes/genes contribute to the connections across all those for that drug/drug family.

5. The data for the heat map (as displayed on the screen) is also downloadable as nFractions.csv/pFractions.csv from the url, http://localhost:8090/download
