# Supplementary material for: QUADrATiC: scalable gene expression connectivity mapping for repurposing FDA-approved therapeutics
Source: BMC Bioinformatics. 2016 May 4;17:198. doi: 10.1186/s12859-016-1062-1 (PMC4855472; doi:10.1186/s12859-016-1062-1)
Supplement: Additional file 6 — The output from Qiagen IPA for the contributing genes for the top 5 drug connections. (PDF 2068.48 kb) [file 12859_2016_1062_MOESM6_ESM.pdf]

Amiodarone

Canonical Pathways

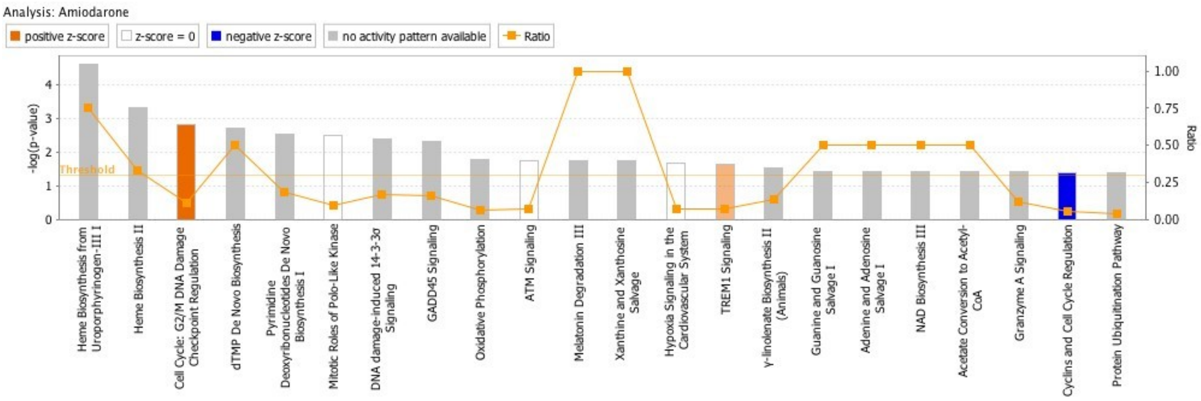

© 2000-2016 QIAGEN. All rights reserved.

Diseases & Functions

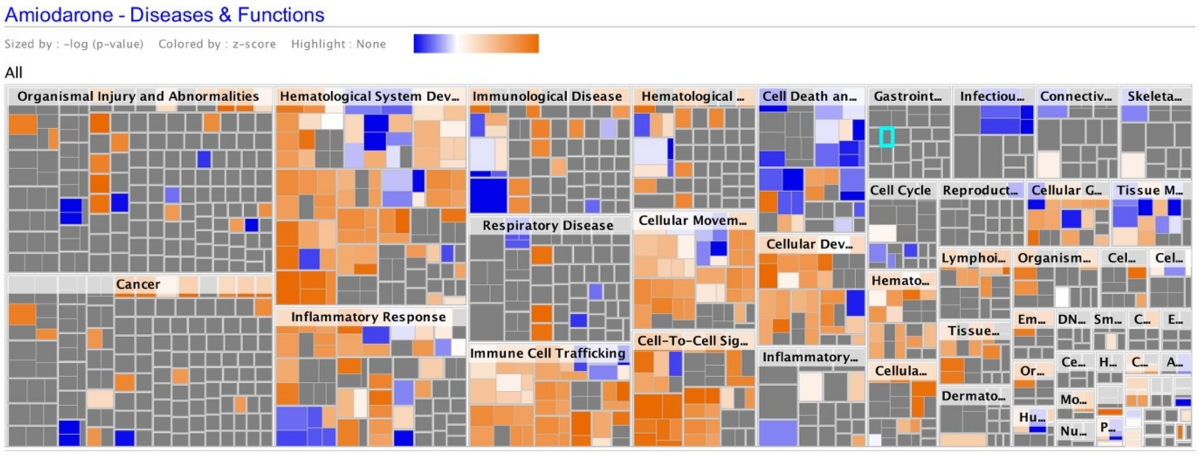

© 2000-2016 QIAGEN. All rights reserved.

Networks

© 2000-2016 QIAGEN. All rights reserved.

ID	Molecules in Network	Score	Focus Molecules	Top Diseases and Functions
1	Akt,alcohol group acceptor phosphotransferase,APC (complex),CCNE2,CDC20,CDK1,Cyclin A,Cyclin B,Cyclin E,DHFR,DLGAP5,DLK1,E2f,E2F8,GMNN,HIST3H2A,IGFBP7,LAPTM4B,MAD2L1,MAK,NUSAP1,PBK,PLK4,PRC1,RACGAP1,Rb,RRM2,TCL1A,TFDP2,TFRC,TTK,TYMS,UBE2,UBE2C,UBE2S	48	26	Cell Cycle, Cell Death and Survival, Organismal Development
2	ADAM8,B-cell receptor,BCL6,BCL11B,BPI,BTG3,CASP4,CCNB2,CD79A,CENPU,CKS2,CLEC10A,CLEC11A,HIP1,HLA-DR,IFN alpha/beta,Igg3,Ikb,IL-1R,IL13RA1,IL1R2,IL1RAP,KDM2A,KDM6B,NFkB (complex),OLFM4,PAX5,PGLYRP1,POU2AF1,SMC4,TH2 Cytokine,Tlr,TLR5,TNFRSF10C,TREM1	48	26	Hematological System Development and Function, Tissue Morphology, Cancer
3	ADRB,ARG1,ARL4A,AZU1,CaMKII,CENPF,COPS2,Creb,CREB5,DTL,ERMAP,H1F0,HIST1H1C,HIST1H4C,Histone H1,Histone h3,KCNJ2,KIAA0101,KIAA0226L,LEF1,MME,NRBF2,NREP,PCNA,Pkc(s),Rab5,Rac,RARA,RBX1,TCF,TLE3,TSEN34,UBE2D1,Ubiquitin,WLS	46	25	Digestive System Development and Function, Embryonic Development, Organ Development
4	Alpha 1 antitrypsin,CAMP,CEACAM6,CEACAM8,chymotrypsin,CTSG,DEFA4,DEFA3 (includes others),Ecm,ELANE,elastase,ERK1/2,FCGR3A/FCGR3B,GK,GYPA,Hnp alpha,HP,ITGAX,LITAF,LTF,MMP25,MPO,Mucin,NUCB2,Par,PELI1,PI3,	43	24	Infectious Diseases, Cell Death and Survival, Respiratory Disease

	Pro-inflammatory Cytokine,PRTN3,SERPINB10,signal peptidase,TCN1,trypsin,UROD,XK			
5	Alpha tubulin,AQP1,BCR (complex),CA2,caspase,CD24,COX7B,CYB5A,cytochrome C,cytochrome-c oxidase,ERK,FABP5,FSCN1,GDF15,H2AFX,HIST1H2BC,HMMR,IgD,Igm,MAP2K1/2,MCL1,Mek,MIR101,NDUFA4,OSBPL10,Pak,PRDX2,Raf,RPS27L,SLC16A1,Sos,STMN1,TIP60,TOP2A,ZFP36L1	33	20	Molecular Transport, Cancer, Organismal Injury and Abnormalities
6	ATP8B4,BCAP29,CCDC8,CITED2,COPS5,CPNE3,CPOX,CREB3,CREB3L2,DEK,ELAVL1,FAM129A,GAPVD1,GAS2L3,HEATR5B,IARS,IL13,ISCA1,LARP1,LITAF,MAP3K7CL,MGST3,PLCXD1,PPOX,RBM47,RIN2,RPRM,SLC25A44,SMPD2,SNRPG,SUMF2,TMEM140,TMEM14B,TMEM30A,TOR1AIP1	25	16	Small Molecule Biochemistry, Gastrointestinal Disease, Hematological Disease
7	Actin,ADM,Cofilin,Collagen type I,Collagen(s),DOCK4,F Actin,GBP2,GCLM,Growth hormone,GYPB,GYPE,HBZ,hemoglobin,Il8r,Ldh (complex),LDL,LYN,MARCKS,MYB,NADPH oxidase,NAMPT,Notch,Pdgf (complex),PDGF BB,PI3K (complex),PI3K (family),PP2A,Rap1,RGCC,SNRPG,SORL1,SPTA1,STAT5a/b,TPM1	25	16	Cell Morphology, Organismal Injury and Abnormalities, Renal and Urological Disease
8	AES,AP2M1,AP5Z1,APP,ATG7,CCNJL,CDC42EP2,CEACAM6,CENPT,CMAS,CYTH4,DSN1,DUT,ESR1,FCHO2,GABARAP,GTPase,Iga,MIR22HG,MITF,MND1,NEDD4,RUNDC3A,SPC24,SPC25,SPECC1L,TBCD,TBRG1,TCP11L1,TECPR2,TMCC2,TP73,UBL3,UQCRB,UQCRQ	20	14	Cancer, Organismal Injury and Abnormalities, Gastrointestinal Disease

9	Adaptor protein 2,ARHGAP26,CCR7,chemokine,chemokine receptor,Clathrin,CXCR1,CXCR2,F2RL1,FFAR2,Focal adhesion kinase,G protein alphai,Gi-coupled receptor,Gpcr,HCAR3,IgG1,IgG2b,IGHM,IL12 (family),Mapk,Metalloprotease,NMDA Receptor,NUMB,P110,p85 (pik3r),PTAFR,Ras,Ras homolog,RNASE2,S1PR1,SELPLG,Sfk,Shc,SRC (family),tubulin (complex)	19	13	Cellular Movement, Hematological System Development and Function, Immune Cell Trafficking
10	26s Proteasome,Alp,ALPL,AMPK,CDKN2C,Ck2,collagen,DNAJB4,DUSP1,estrogen receptor,GATA3,HDL,HSP,Hsp70,Hsp90,HSPA6,Ifn,IFN Beta,IgG,IL1,Interferon alpha,KCNH2,LCN2,LOC254896,MHC Class II (complex),Mmp,Nos,P38 MAPK,POLR2K,RAD51AP1,RNA polymerase II,SECTM1,TAL1,Tnf (family),Vegf	19	13	Cancer, Gastrointestinal Disease, Organismal Injury and Abnormalities
11	ACSL1,Alpha catenin,Ap1,AQP9,BCL3,C/ebp,Collagen Alpha1,CYTIP,DAAM1,Fc gamma receptor,Fcer1,Fibrinogen,FNBP1L,Gm-csf,Ige,IgG2a,IL12 (complex),IL4R,Immunoglobulin,Jnk,KLHL2,MS4A3,N-cor,Nfat (family),Nr1h,PNP,Rar,RNASE3,Rxr,Smad2/3,SYK/ZAP,Tgf beta,TGM2,THBS1,WIPF1	18	14	Cellular Function and Maintenance, Hematological System Development and Function, Post-Translational Modification
12	AKIRIN2,ANP32B,ANP32E,ASPRV1,CLEC5A,CYP1A1,CYP2D6,EDEM3,EPHX1,HEMGN,HEXA,HIST1H4B,HLA-J,HNF4A,HYI,iron,KCNK9,LRRC8C,LSM5,MAN1C1,MINPP1,PPT1,PRG2,PTP4A1,REXO2,RHAG,RHCE/R	17	12	Cardiovascular Disease, Cell Death and Survival, Cellular Assembly and Organization

	HD,SMARCA4,TNF,TPP2,TSPO2,UCHL5,ULBP2,WBSCR22,ZFP64			
13	AURKA,Calmodulin,Cbp/p300,CD3,Cg,Collagen type IV,FSH,Granzyme,Gsk3,H2AFV,HIST1H4J,HISTONE,Histone h4,ICAM4,Il31,Insulin,Integrin,KCNJ15,Lh,MAPRE2,MCM4,Pka,PLC,PTX3,RAB13,SEC14L1,SEPP1,STOM,SYT9,TCR,TLK1,TUBB,TUBG1,UQCC2,Wnt	16	12	Cancer, Organismal Injury and Abnormalities, Respiratory Disease
14	A1BG,CRISP3,ELF5,KNG1	1	1	Cancer, Gastrointestinal Disease, Hepatic System Disease

Haematological System Development & Function Network

Network 2 : Amiodarone : PMFvsPVDrugSignatureActions : Amiodarone

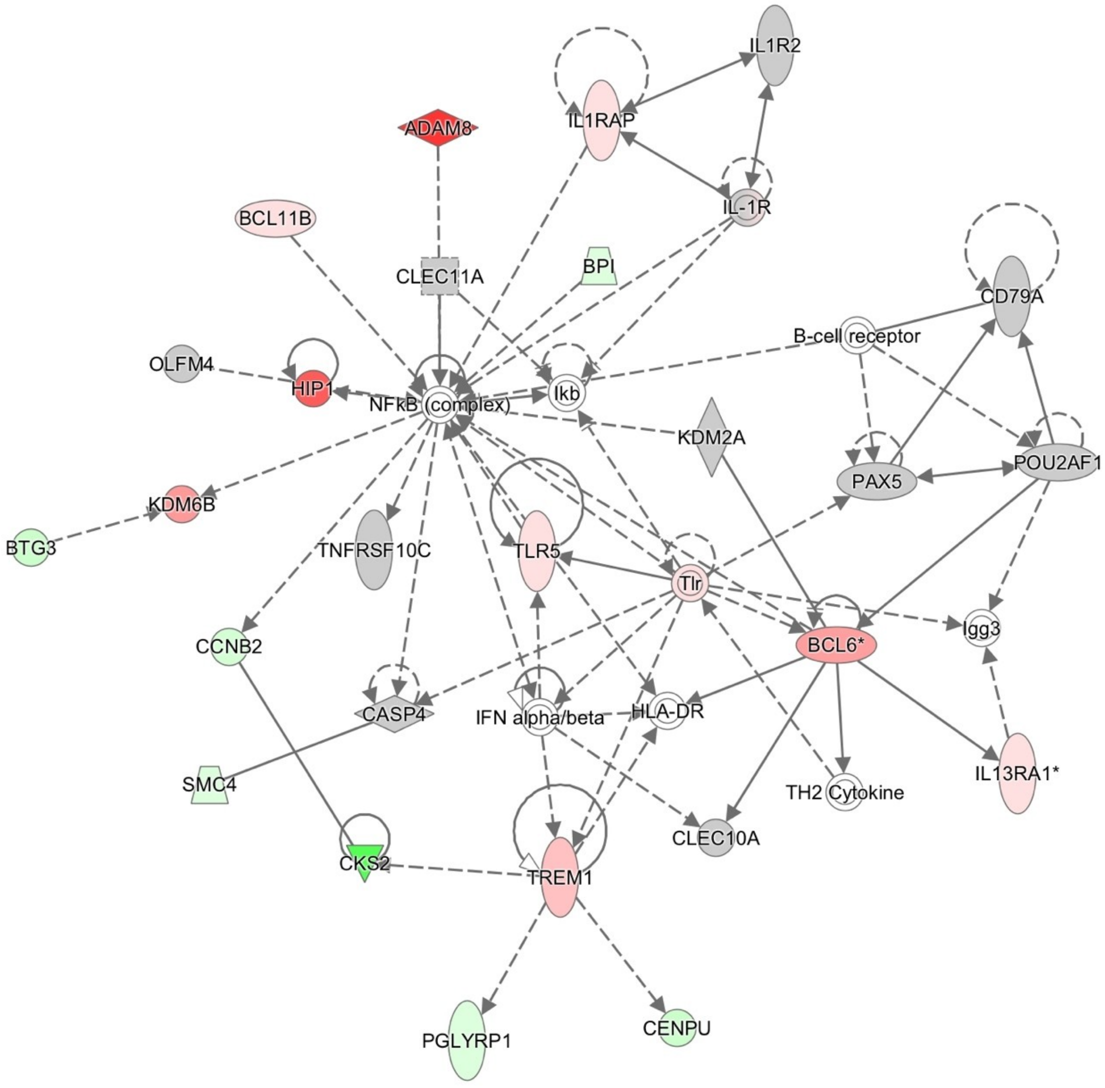

© 2000-2016 QIAGEN. All rights reserved.

Pentamidine

Canonical Pathways

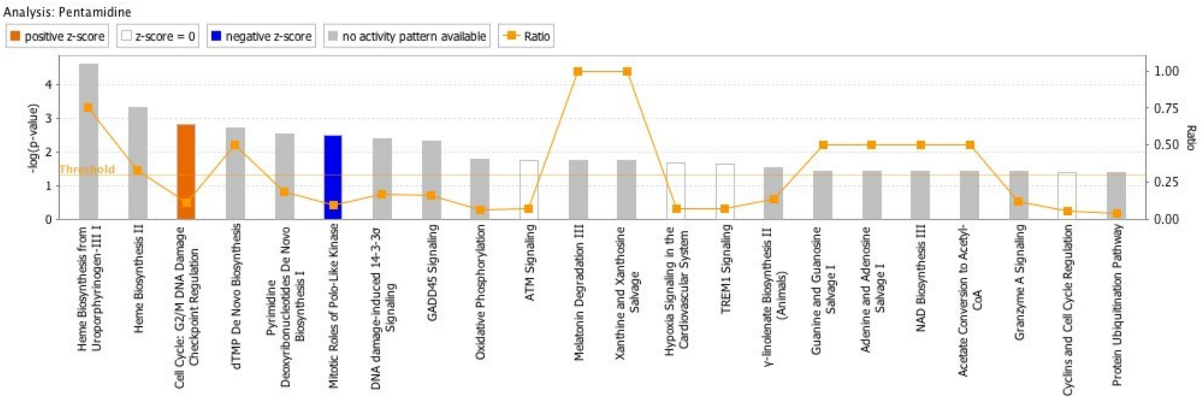

© 2000-2016 QIAGEN. All rights reserved.

Diseases & Functions

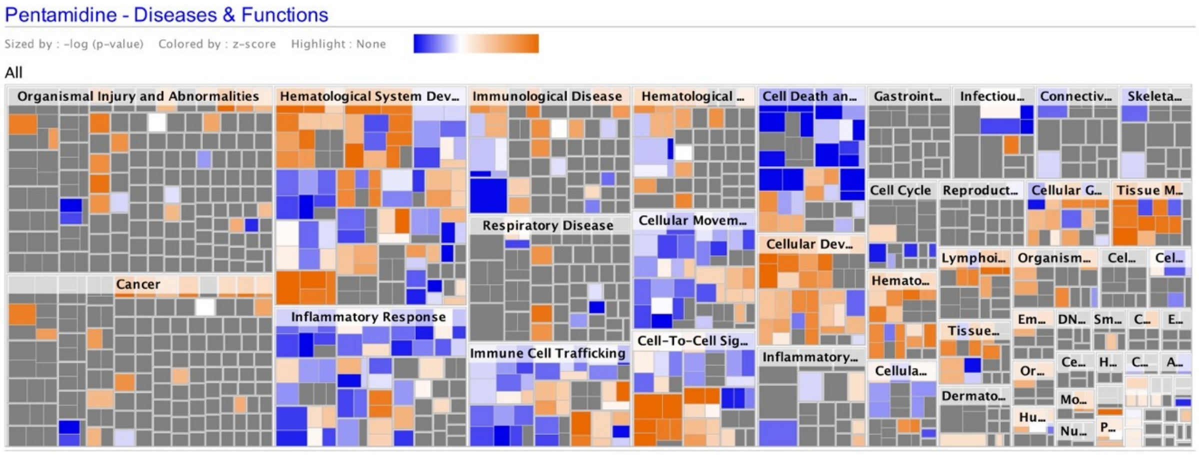

© 2000-2016 QIAGEN. All rights reserved.

Networks

© 2000-2016 QIAGEN. All rights reserved.

ID	Molecules in Network	Score	Focus Molecules	Top Diseases and Functions
1	Akt,alcohol group acceptor phosphotransferase,APC (complex),CCNE2,CDC20,CDK1,Cyclin A,Cyclin B,Cyclin E,DHFR,DLGAP5,DLK1,E2f,E2F8,GMNN,HIST3H2A,IGFBP7,LAPTM4B,MAD2L1,MAK,NUSAP1,PBK,PLK4,PRC1,RACGAP1,Rb,RRM2,TCL1A,TFDP2,TFRC,TTK,TYMS,UBE2,UBE2C,UBE2S	48	26	Cell Cycle, Cell Death and Survival, Organismal Development
2	ADAM8,B-cell receptor,BCL6,BCL11B,BPI,BTG3,CASP4,CCNB2,CD79A,CENPU,CKS2,CLEC10A,CLEC11A,HIP1,HLA-DR,IFN alpha/beta,Igg3,Ikb,IL-1R,IL13RA1,IL1R2,IL1RAP,KDM2A,KDM6B,NFkB (complex),OLFM4,PAX5,PGLYRP1,POU2AF1,SMC4,TH2 Cytokine,Tlr,TLR5,TNFRSF10C,TREM1	48	26	Hematological System Development and Function, Tissue Morphology, Inflammatory Disease
3	ADRB,ARG1,ARL4A,AZU1,CaMKII,CENPF,COPS2,Creb,CREB5,DTL,ERMAP,H1F0,HIST1H1C,HIST1H4C,Histone H1,Histone h3,KCNJ2,KIAA0101,KIAA0226L,LEF1,MME,NRBF2,NREP,PCNA,Pkc(s),Rab5,Rac,RARA,RBX1,TCF,TLE3,TSEN34,UBE2D1,Ubiquitin,WLS	46	25	Gene Expression, Cancer, Gastrointestinal Disease
4	Alpha 1	43	24	Infectious Diseases, Respiratory

	antitrypsin,CAMP,CEACAM6,CEACAM8,chymotrypsin,CTSG,DEFA4,DEFA3 (includes others),Ecm,ELANE,elastase,ERK1/2,FCGR3A/FCGR3B,GK,GYPA,Hnp alpha,HP,ITGAX,LITAF,LTF,MMP25,MPO,Mucin,NUCB2,Par,PELI1,PI3,Pro-inflammatory Cytokine,PRTN3,SERPINB10,signal peptidase,TCN1,trypsin,UROD,XK			Disease, Inflammatory Disease
5	Alpha tubulin,AQP1,BCR (complex),CA2,caspase,CD24,COX7B,CYB5A,cytochrome C,cytochrome-c oxidase,ERK,FABP5,FSCN1,GDF15,H2AFX,HIST1H2BC,HMMR,IgD,Igm,MAP2K1/2,MCL1,Mek,MIR101,NDUFA4,OSBPL10,Pak,PRDX2,Raf,RPS27L,SLC16A1,Sos,STMN1,TIP60,TOP2A,ZFP36L1	33	20	Molecular Transport, Cancer, Organismal Injury and Abnormalities
6	ATP8B4,BCAP29,C18orf25,CCDC8,CITED2,COPS5,CPNE3,CPOX,CREB3,CREB3L2,ELAVL1,FAM129A,FBXO11,GAS2L3,HEATR5B,IL13,IL13RA1,ISCA1,LITAF,MAP3K7CL,MGST3,NCLN,NLRX1,NREP,PPOX,RBM47,RPRM,SERPINB4,SLC25A44,SUMF2,TIA1,TMEM140,TMEM14B,TMEM30A,TOR1AIP1	27	17	Cancer, Organismal Injury and Abnormalities, Developmental Disorder
7	Actin,ADM,Cofilin,Collagen type I,Collagen(s),DOCK4,F Actin,GBP2,GCLM,Growth hormone,GYPB,GYPE,HBZ,hemoglobin,Il8r,Ldh (complex),LDL,LYN,MARCKS,MYB,NADPH oxidase,NAMPT,Notch,Pdgf (complex),PDGF BB,PI3K (complex),PI3K (family),PP2A,Rap1,RGCC,SNRPG,SORL1,SPTA1,STAT5a/b,TP	25	16	Cancer, Organismal Injury and Abnormalities, Connective Tissue Disorders

8	M1 AKIRIN2,ANP32E,BMPER,CARD16,catechol,CCR8,CLEC5A,CYP1A1,CYP2D6,EDEM3,HIST1H4B,HLA-J,HNF4A,HYI,iron,KCNK9,KIF20A,LSM5,MAN1C1,MINPP1,PRG2,REXO2,RHAG,RHCE/RHD,RNASE2,SMARCA4,TNF,TPP2,TSPO2,TYMS,UCHL5,WBSCR22,WNT10A,ZFP64,ZNF589	21	14	Cardiovascular Disease, Cell Death and Survival, Cellular Assembly and Organization
9	Adaptor protein 2,ARHGAP26,CCR7,chemokine,chemokine receptor,Clathrin,CXCR1,CXCR2,F2RL1,FFAR2,Focal adhesion kinase,G protein alphai,Gi-coupled receptor,Gpcr,HCAR3,IgG1,IgG2b,IGHM,IL12 (family),Mapk,Metalloprotease,NMDA Receptor,NUMB,P110,p85 (pik3r),PTAFR,Ras,Ras homolog,RNASE2,S1PR1,SELPLG,Sfk,Shc,SRC (family),tubulin (complex)	19	13	Cellular Movement, Hematological System Development and Function, Immune Cell Trafficking
10	26s Proteasome,Alp,ALPL,AMPK,CDKN2C,Ck2,collagen,DNAJB4,DUSP1,estrogen receptor,GATA3,HDL,HSP,Hsp70,Hsp90,HSPA6,Ifn,IFN Beta,IgG,IL1,Interferon alpha,KCNH2,LCN2,LOC254896,MHC Class II (complex),Mmp,Nos,P38 MAPK,POLR2K,RAD51AP1,RNA polymerase II,SECTM1,TAL1,Tnf (family),Vegf	19	13	Cancer, Gastrointestinal Disease, Organismal Injury and Abnormalities
11	ACSL1,Alpha catenin,Ap1,AQP9,BCL3,C/ebp,Collagen Alpha1,CYTIP,DAAM1,Fc gamma receptor,Fcer1,Fibrinogen,FNB	18	14	Cellular Function and Maintenance, Hematological System Development and Function, Post-Translational Modification

	P1L,Gm-csf,Ige,IgG2a,IL12 (complex),IL4R,Immunoglobulin,Jnk,KLHL2,MS4A3,N-cor,Nfat (family),Nr1h,PNP,Rar,RNASE3,Rxr,Smad2/3,SYK/ZAP,Tgf beta,TGM2,THBS1,WIPF1			
12	AES,AP2M1,AP5Z1,APP,ATG7,C11orf63,CCNJL,CMAS,CYTH4,DIEXF,DUT,ERMN,ESR1,GABARAP,GTPase,Iga,MIR22HG,MITF,MND1,NEDD4,PRR16,RASGEF1A,RUNDC3A,SP140L,SPC25,TCP11L1,TECPR2,TMCC2,TP73,TRIM52,UBL3,UQCRB,UQCRH,UQCRHL,UQCRQ	18	13	Developmental Disorder, Hereditary Disorder, Metabolic Disease
13	ARHGAP35,AURKA,Calmodulin,Cbp/p300,CCR8,CD3,Cg,Collagen type IV,FSH,Granzyme,Gsk3,HIST1H4J,HISTONE,Histone h4,ICAM4,Il31,Insulin,Integrin,KCNJ15,Lh,MCM4,PFDN4,Pka,PLC,PRMT7,PTX3,RAB13,RNU7-1,SEC14L1,SEPP1,STOM,TCR,TUBB,TUBG1,Wnt	16	12	Cancer, Gastrointestinal Disease, Organismal Injury and Abnormalities
14	A1BG,CRISP3,ELF5,KNG1	1	1	Cancer, Gastrointestinal Disease, Hepatic System Disease

Haematological System Development & Function Network

Network 2 : Pentamidine : PMFvsPVDrugSignatureActions : Pentamidine

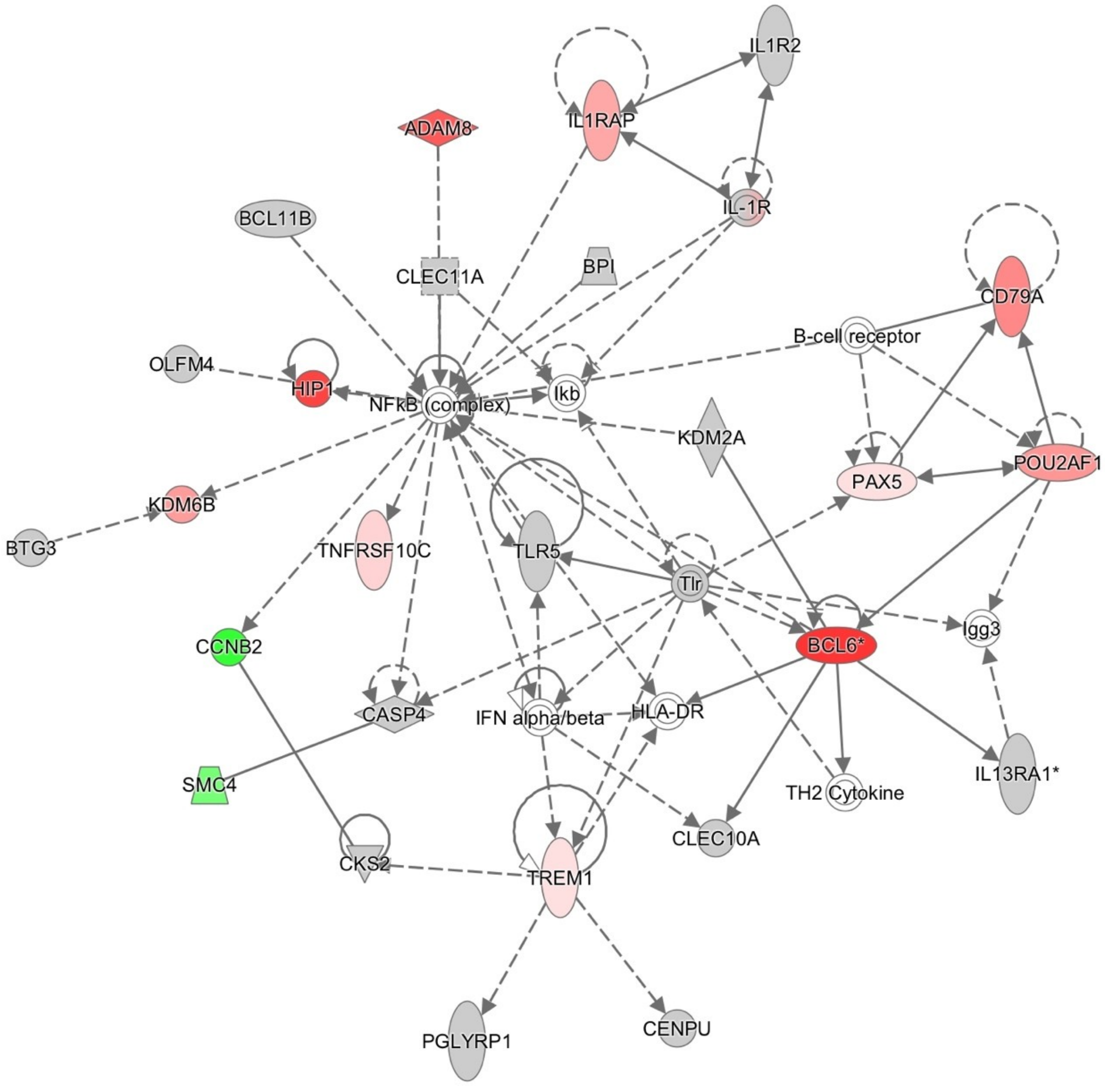

© 2000-2016 QIAGEN. All rights reserved.

Azacitidine

Canonical Pathways

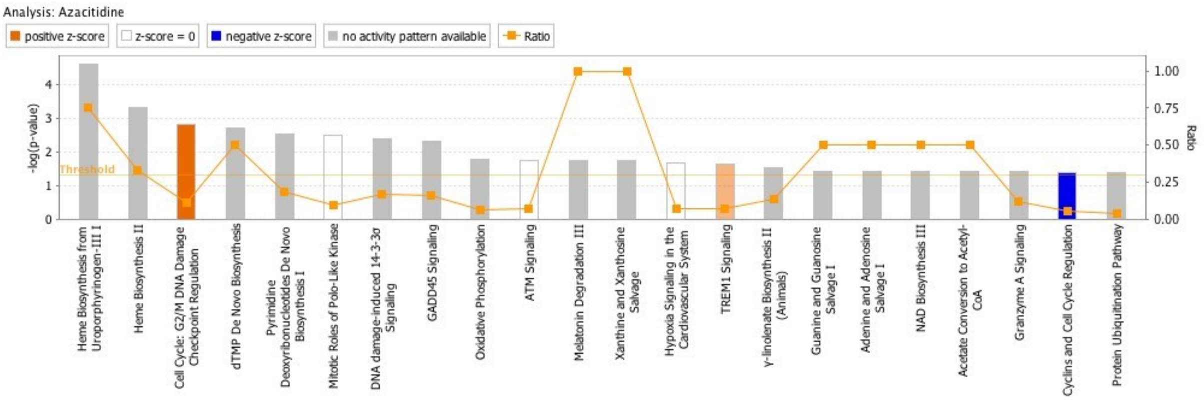

© 2000-2016 QIAGEN. All rights reserved.

Diseases & Functions

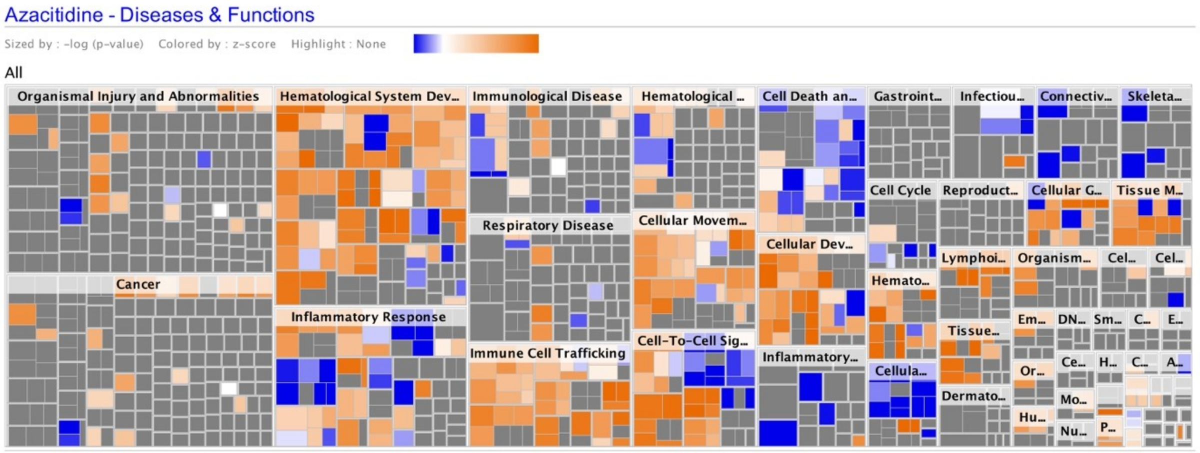

© 2000-2016 QIAGEN. All rights reserved.

Networks

© 2000-2016 QIAGEN. All rights reserved.

ID	Molecules in Network	Score	Focus Molecules	Top Diseases and Functions
1	Akt,alcohol group acceptor phosphotransferase,APC (complex),CCNE2,CDC20,CDK1,Cyclin A,Cyclin B,Cyclin E,DHFR,DLGAP5,DLK1,E2f,E2F8,GMNN,HIST3H2A,IGFBP7,LAPTM4B,MAD2L1,MAK,NUSAP1,PBK,PLK4,PRC1,RACGAP1,Rb,RRM2,TCL1A,TFDP2,TFRC,TTK,TYMS,UBE2,UBE2C,UBE2S	48	26	Cell Cycle, Cell Death and Survival, Organismal Development
2	ADAM8,B-cell receptor,BCL6,BCL11B,BPI,BTG3,CASP4,CCNB2,CD79A,CENPU,CKS2,CLEC10A,CLEC11A,HIP1,HLA-DR,IFN alpha/beta,Igg3,Ikb,IL-1R,IL13RA1,IL1R2,IL1RAP,KDM2A,KDM6B,NFkB (complex),OLFM4,PAX5,PGLYRP1,POU2AF1,SMC4,TH2 Cytokine,Tlr,TLR5,TNFRSF10C,TREM1	48	26	Hematological System Development and Function, Tissue Morphology, Inflammatory Disease
3	ADRB,ARG1,ARL4A,AZU1,CaMKII,CENPF,COPS2,Creb,CREB5,DTL,ERMAP,H1F0,HIST1H1C,HIST1H4C,Histone H1,Histone h3,KCNJ2,KIAA0101,KIAA0226L,LEF1,MME,NRBF2,NREP,PCNA,Pkc(s),Rab5,Rac,RARA,RBX1,TCF,TLE3,TSEN34,UBE2D1,Ubiquitin,WLS	46	25	Digestive System Development and Function, Embryonic Development, Organ Development
4	Alpha 1 antitrypsin,CAMP,CEACAM6,CEACAM8,chymotrypsin,CTSG,DEFA4,DEFA3 (includes others),Ecm,ELANE,elastase,ERK1/2,FCGR3A/FCGR3B,GK,GYPA,Hnp alpha,HP,ITGAX,LITAF,LTF,MMP25,MPO,Mucin,NUCB2,Par,PELI1,PI3,Pro-inflammatory	43	24	Infectious Diseases, Cancer, Organismal Injury and Abnormalities

	Cytokine,PRTN3,SERPINB10,signal peptidase,TCN1,trypsin,UROD,XK			
5	Alpha tubulin,AQP1,BCR (complex),CA2,caspase,CD24,COX7B,CYB5A,cytochrome C,cytochrome-c oxidase,ERK,FABP5,FSCN1,GDF15,H2AFX,HIST1H2BC,HMMR,IgD,Igm,MAP2K1/2,MCL1,Mek,MIR101,NDUFA4,OSBPL10,Pak,PRDX2,Raf,RPS27L,SLC16A1,Sos,STMN1,TIP60,TOP2A,ZFP36L1	33	20	Molecular Transport, Cancer, Organismal Injury and Abnormalities
6	ADAM28,ATP8B4,BCAP29,CCDC8,CITED2,COPS5,CPNE3,CPOX,CREB3,CREB3L2,ELAVL1,FAM129A,HEATR5B,IL13,IL13RA1,IL1R2,ISCA1,MAP3K7CL,MGST3,NCLN,NREP,PLAC8,PLCXD1,PPOX,RBM47,RIN2,RNF138,SEH1L,SLC25A44,SPRR1A,TMEM140,TMEM14B,TMEM30A,TOR1AIP1,ZNRF2	27	17	Developmental Disorder, Hereditary Disorder, Neurological Disease
7	Actin,ADM,Cofilin,Collagen type I,Collagen(s),DOCK4,F Actin,GBP2,GCLM,Growth hormone,GYPB,GYPE,HBZ,hemoglobin,Il8r,Ldh (complex),LDL,LYN,MARCKS,MYB,NADPH oxidase,NAMPT,Notch,Pdgf (complex),PDGF BB,PI3K (complex),PI3K (family),PP2A,Rap1,RGCC,SNRPG,SORL1,SPTA1,STAT5a/b,TPM1	25	16	Cell Morphology, Organismal Injury and Abnormalities, Renal and Urological Disease
8	AK2,AKIRIN2,ANP32E,AQP9,ASPRV1,BMPER,catechol,CLEC5A,CYP1A1,EDEM3,HIST1H4B,HLA-J,HNF4A,HYI,iron,KCNK9,LSM5,MAN1C1,MARCH1,MINPP1,MTX3,NEK7,PRG2,Prl2c2 (includes others),REXO2,RHAG,RHCE/RHD,SMARCA4,TNF,TPP2,TSPO2,TYMS,UCHL5,ZFP64,ZNF503	21	14	Cardiovascular System Development and Function, Cellular Function and Maintenance, Tissue Development
9	Adaptor protein 2,ARHGAP26,CCR7,chemokine,chemokine receptor,Clathrin,CXCR1,CXCR2,F2RL1,FFAR2,Focal adhesion kinase,G protein alphai,Gi-coupled receptor,Gpcr,HCAR3,IgG1,IgG2b,IGHM,IL12 (family),Mapk,Metalloprotease,NMDA	19	13	Cellular Movement, Hematological System Development and Function, Immune Cell Trafficking

	Receptor,NUMB,P110,p85 (pik3r),PTAFR,Ras,Ras homolog,RNASE2,S1PR1,SELPLG,Sfk,Shc ,SRC (family),tubulin (complex)			
10	26s Proteasome,Alp,ALPL,AMPK,CDKN2C,Ck 2,collagen,DNAJB4,DUSP1,estrogen receptor,GATA3,HDL,HSP,Hsp70,Hsp90,H SPA6,Ifn,IFN Beta,IgG,IL1,Interferon alpha,KCNH2,LCN2,LOC254896,MHC Class II (complex),Mmp,Nos,P38 MAPK,POLR2K,RAD51AP1,RNA polymerase II,SECTM1,TAL1,Tnf (family),Vegf	19	13	Cancer, Gastrointestinal Disease, Organismal Injury and Abnormalities
11	AURKA,BTG3,Calmodulin,Cbp/p300,CD3 ,Cg,Collagen type IV,FSH,GPRC5A,Gsk3,H2AFV,HIST1H4J,HI STONE,Histone h4,ICAM4,Il31,Insulin,Integrin,KCNJ15,L h,MCM4,Pka,PLC,PTX3,RAB13,SCO1,SEC 14L1,SEPP1,STOM,SYT9,TCR,TPST1,TUBB ,TUBG1,Wnt	18	13	Cancer, Gastrointestinal Disease, Organismal Injury and Abnormalities
12	ACSL1,Alpha catenin,Ap1,AQP9,BCL3,C/ebp,Collagen Alpha1,CYTIP,DAAM1,Fc gamma receptor,Fcer1,Fibrinogen,FNBP1L,Gm-csf,Ige,IgG2a,IL12 (complex),IL4R,Immunoglobulin,Jnk,KLH L2,MS4A3,N-cor,Nfat (family),Nr1h,PNP,Rar,RNASE3,Rxr,Smad 2/3,SYK/ZAP,Tgf beta,TGM2,THBS1,WIPF1	18	14	Cellular Function and Maintenance, Hematological System Development and Function, Post-Translational Modification
13	AES,ANXA9,AP2M1,AP5Z1,APP,ATG7,C1 1orf63,CCNJL,CENPQ,CMAS,CYTH4,DUT, ERMN,ESR1,GABARAP,GTPase,HMCES,Ig a,MIR22HG,MITF,NEDD4,PRR16,RASGEF 1A,RASL11B,RUNDC3A,SP140L,SPANXN 3,SPC25,TCP11L1,TECPR2,TMCC2,TP73,T RIM52,UQCRB,UQCRQ	18	13	Developmental Disorder, Hereditary Disorder, Metabolic Disease
14	A1BG,CRISP3,ELF5,KNG1	1	1	Cancer, Gastrointestinal Disease, Hepatic System Disease

Haematological System Development & Function Network

Network 2 : Azacitidine : PMFvsPVDrugSignatureActions : Azacitidine

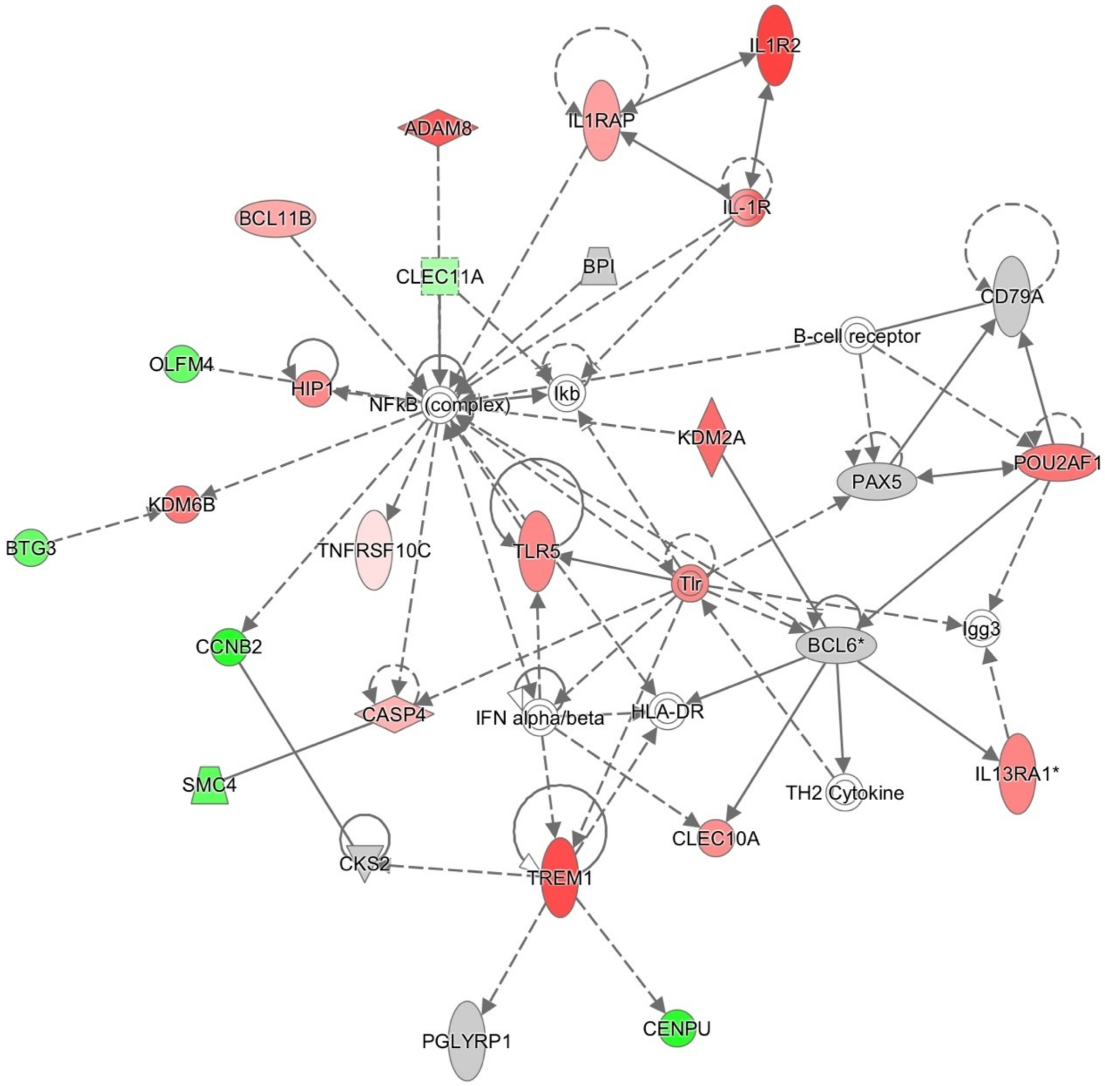

© 2000-2016 QIAGEN. All rights reserved.

Pemetrexed

Canonical Pathways

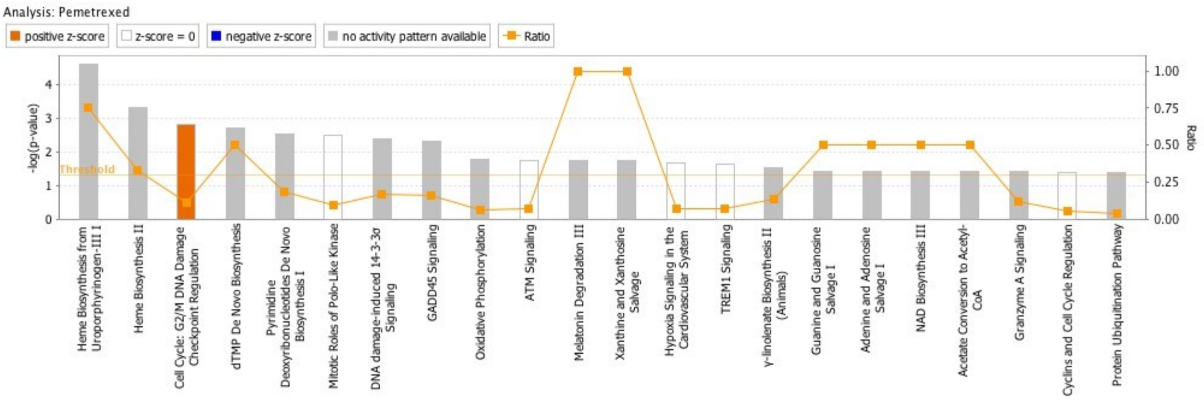

© 2000-2016 QIAGEN. All rights reserved.

Diseases & Functions

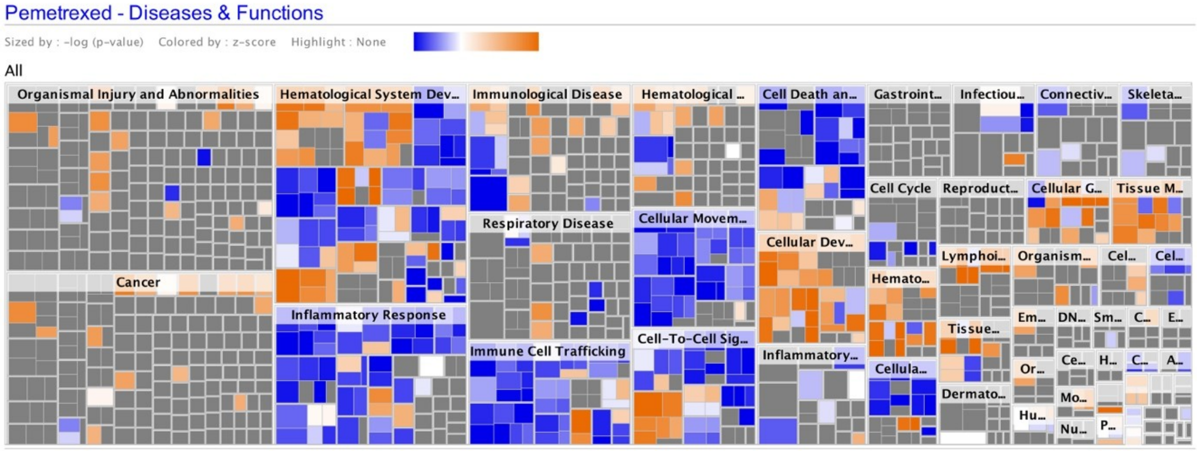

© 2000-2016 QIAGEN. All rights reserved.

Networks

© 2000-2016 QIAGEN. All rights reserved.

ID	Molecules in Network	Score	Focus Molecules	Top Diseases and Functions
1	Akt,alcohol group acceptor phosphotransferase,APC (complex),CCNE2,CDC20,CDK1,Cyclin A,Cyclin B,Cyclin E,DHFR,DLGAP5,DLK1,E2f,E2F8,GMNN,HIST3H2A,IGFBP7,LAPTM4B,MAD2L1,MAK,NUSAP1,PBK,PLK4,PRC1,RACGAP1,Rb,RRM2,TCL1A,TFDP2,TFRC,TTK,TYMS,UBE2,UBE2C,UBE2S	48	26	Cell Cycle, Cellular Growth and Proliferation, DNA Replication, Recombination, and Repair
2	ADAM8,B-cell receptor,BCL6,BCL11B,BPI,BTG3,CASP4,CCNB2,CD79A,CENPU,CKS2,CLEC10A,CLEC11A,HIP1,HLA-DR,IFN alpha/beta,Igg3,Ikb,IL-1R,IL13RA1,IL1R2,IL1RAP,KDM2A,KDM6B,NFkB (complex),OLFM4,PAX5,PGLYRP1,POU2AF1,SMC4,TH2 Cytokine,Tlr,TLR5,TNFRSF10C,TREM1	48	26	Cancer, Organismal Injury and Abnormalities, Cell Death and Survival
3	ADRB,ARG1,ARL4A,AZU1,CaMKII,CENPF,COPS2,Creb,CREB5,DTL,ERMAP,H1F0,HIST1H1C,HIST1H4C,Histone H1,Histone h3,KCNJ2,KIAA0101,KIAA0226L,LEF1,MME,NRBF2,NREP,PCNA,Pkc(s),Rab5,Rac,RARA,RBX1,TCF,TLE3,TSEN34,UBE2D1,Ubiquitin,WLS	46	25	Digestive System Development and Function, Embryonic Development, Organ Development
4	Alpha 1 antitrypsin,CAMP,CEACAM6,CEACAM8,chymotrypsin,CTSG,DEFA4,DEFA3 (includes others),Ecm,ELANE,elastase,ERK1/2,FCGR3A/FCGR3B,GK,GYPA,Hnp alpha,HP,ITGAX,LITAF,LTF,MMP25,MPO,Mucin,NUCB2,Par,PELI1,PI3,Pro-inflammatory	43	24	Infectious Diseases, Cancer, Organismal Injury and Abnormalities

	Cytokine,PRTN3,SERPINB10,signal peptidase,TCN1,trypsin,UROD,XK			
5	Alpha tubulin,AQP1,BCR (complex),CA2,caspase,CD24,COX7B,CYB5A,cytochrome C,cytochrome-c oxidase,ERK,FABP5,FSCN1,GDF15,H2AFX,HIST1H2BC,HMMR,IgD,Igm,MAP2K1/2,MCL1,Mek,MIR101,NDUFA4,OSBPL10,Pak,PRDX2,Raf,RPS27L,SLC16A1,Sos,STMN1,TIP60,TOP2A,ZFP36L1	33	20	Molecular Transport, Cancer, Organismal Injury and Abnormalities
6	ACVR2B,ARG1,ATP8B4,BCAP29,CCDC8,CITED2,COPS5,CPNE3,CPOX,CREB3,ELAVL1,FAM129A,HEATR5B,IL13,IL1R2,ISCA1,MAP3K7CL,MGST3,NCLN,NLRX1,PAPPA,PLCXD1,PPOX,RBM47,SERPINB4,SLC25A44,SPRR1A,STX7,SUMF2,TMEM140,TMEM14B,TMEM30A,TOR1AIP1,ZC3H14,ZYG11B	25	16	Cardiovascular Disease, Organismal Injury and Abnormalities, Small Molecule Biochemistry
7	Actin,ADM,Cofilin,Collagen type I,Collagen(s),DOCK4,F Actin,GBP2,GCLM,Growth hormone,GYPB,GYPE,HBZ,hemoglobin,Il8r,Ldh (complex),LDL,LYN,MARCKS,MYB,NADPH oxidase,NAMPT,Notch,Pdgf (complex),PDGF BB,PI3K (complex),PI3K (family),PP2A,Rap1,RGCC,SNRPG,SORL1,SPTA1,STAT5a/b,TPM1	25	16	Cell Morphology, Organismal Injury and Abnormalities, Renal and Urological Disease
8	AURKA,AZU1,Calmodulin,Cbp/p300,CD3,Cg,Collagen type IV,DUSP11,FSH,Gsk3,HIST1H4J,HISTONE,Histone h4,HS2ST1,ICAM4,Insulin,Integrin,KCNJ15,Lh,MCM4,PCYOX1,Pka,PLC,PTP4A1,PTX3,RAB13,SEC14L1,SEPP1,SERPINB10,STOM,TCR,TUBB,TUBG1,UNC13D,Wnt	20	14	Cancer, Organismal Injury and Abnormalities, Respiratory Disease
9	AES,AP2M1,AP5Z1,APP,ATG7,C4,CCNJL,CDC42EP2,CEACAM6,CENPQ,CMAS,CYTH4,DIEXF,DUSP11,DUT,ESR1,GABARAP,GBA2,GTPase,Iga,MIR22HG,MITF,MYO15B,NEDD4,RUNDC3A,SPC25,TBRG1,TECPR2,TMCC2,TP73,UQCR10,UQCRB,UQCRH,UQCRHL,UQCRQ	20	14	Neurological Disease, Connective Tissue Development and Function, Skeletal and Muscular System Development and Function
10	Adaptor protein 2,ARHGAP26,CCR7,chemokine,chemoki	19	13	Cellular Movement, Hematological

	ne receptor,Clathrin,CXCR1,CXCR2,F2RL1,FFAR2,Focal adhesion kinase,G protein alphai,Gi-coupled receptor,Gpcr,HCAR3,IgG1,IgG2b,IGHM,IL12 (family),Mapk,Metalloprotease,NMDA Receptor,NUMB,P110,p85 (pik3r),PTAFR,Ras,Ras homolog,RNASE2,S1PR1,SELPLG,Sfk,Shc,SRC (family),tubulin (complex)			System Development and Function, Immune Cell Trafficking
11	ANP32E,ARMC8,catechol,CLEC5A,CYP1A1,DUSP11,EDEM3,FUT11,HIST1H4B,HNF4A,HYI,iron,KCNK9,LSM5,MAEA,MAN1C1,MINPP1,MTX3,N4BP2L2,NEK7,NFRKB,PRG2,PTP4A1,REXO2,RHAG,RHCE/RHD,SEPP1,SMARCA4,TNF,TPP2,TSPO2,UCHL5,ULBP2,YIF1A,ZNF589	19	13	Cancer, Gastrointestinal Disease, Organismal Injury and Abnormalities
12	26s Proteasome,Alp,ALPL,AMPK,CDKN2C,Ck2,collagen,DNAJB4,DUSP1,estrogen receptor,GATA3,HDL,HSP,Hsp70,Hsp90,HSPA6,Ifn,IFN Beta,IgG,IL1,Interferon alpha,KCNH2,LCN2,LOC254896,MHC Class II (complex),Mmp,Nos,P38 MAPK,POLR2K,RAD51AP1,RNA polymerase II,SECTM1,TAL1,Tnf (family),Vegf	19	13	Lymphoid Tissue Structure and Development, Tissue Morphology, Inflammatory Disease
13	ACSL1,Alpha catenin,Ap1,AQP9,BCL3,C/ebp,Collagen Alpha1,CYTIP,DAAM1,Fc gamma receptor,Fcer1,Fibrinogen,FNBP1L,Gm-csf,Ige,IgG2a,IL12 (complex),IL4R,Immunoglobulin,Jnk,KLHL2,MS4A3,N-cor,Nfat (family),Nr1h,PNP,Rar,RNASE3,Rxr,Smad2/3,SYK/ZAP,Tgf beta,TGM2,THBS1,WIPF1	18	14	Cellular Function and Maintenance, Hematological System Development and Function, Post-Translational Modification
14	A1BG,CRISP3,ELF5,KNG1	1	1	Cancer, Gastrointestinal Disease, Hepatic System Disease

Haematological System Development & Function Network

Network 10 : Pemetrexed : PMFvsPVDrugSignatureActions : Pemetrexed

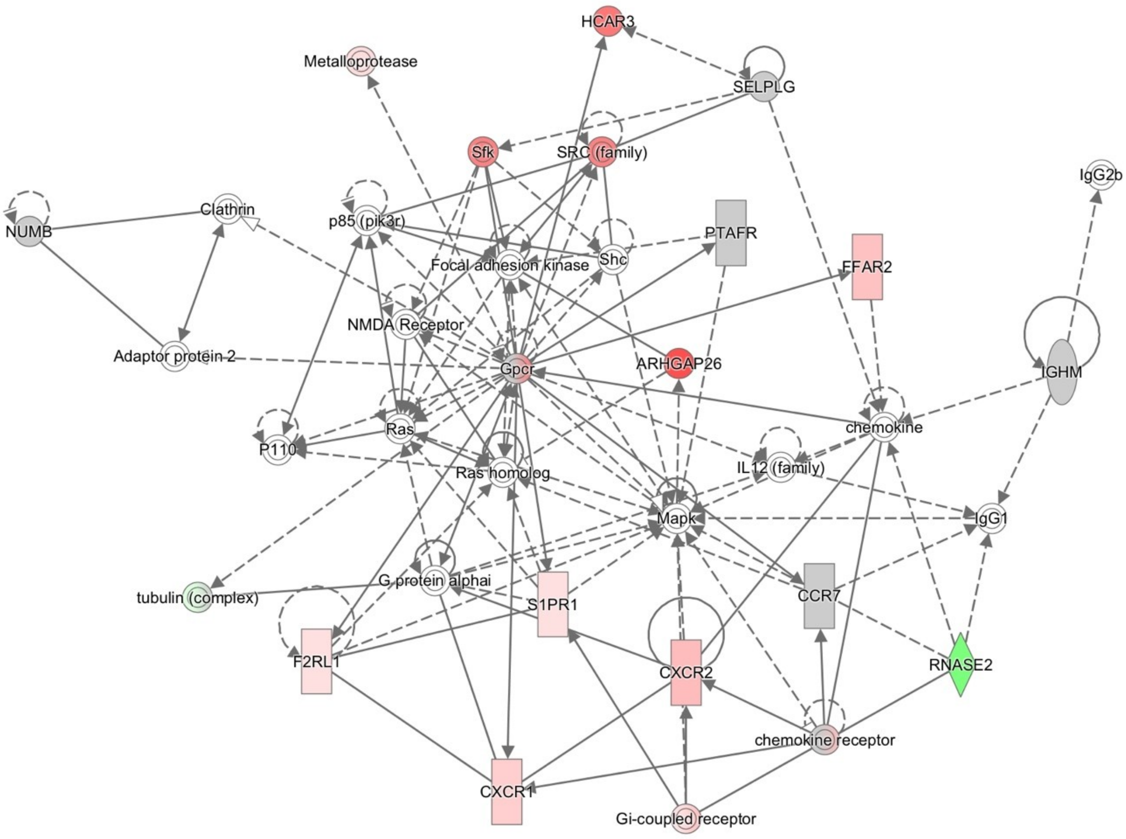

© 2000-2016 QIAGEN. All rights reserved.

Fluocinonide

Canonical Pathways

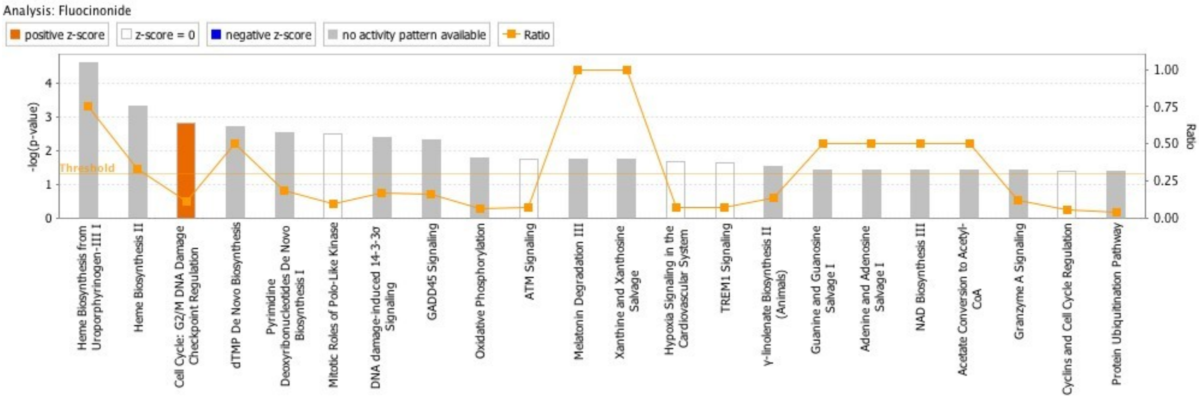

© 2000-2016 QIAGEN. All rights reserved.

Diseases & Functions

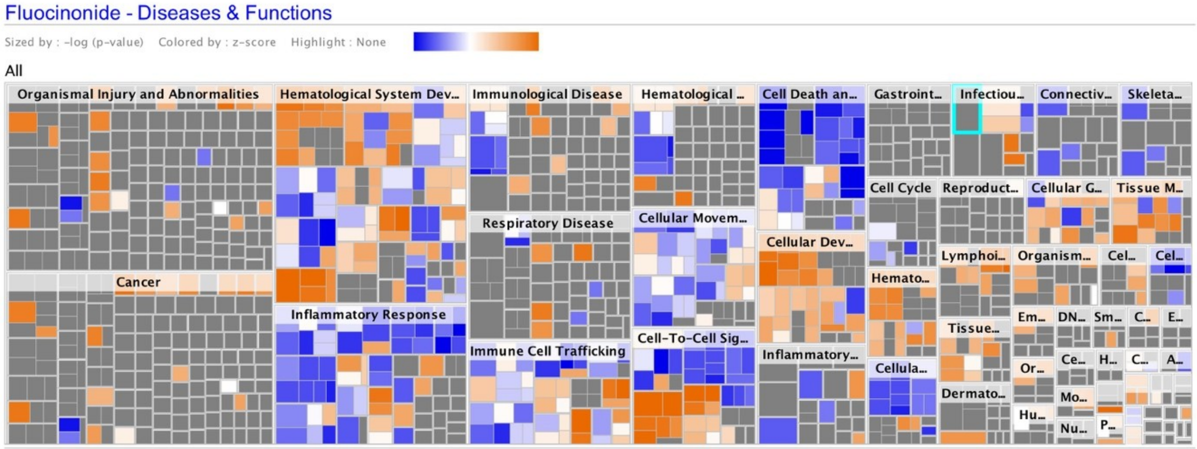

© 2000-2016 QIAGEN. All rights reserved.

Networks

© 2000-2016 QIAGEN. All rights reserved.

ID	Molecules in Network	Score	Focus Molecules	Top Diseases and Functions
1	Akt,alcohol group acceptor phosphotransferase,APC (complex),CCNE2,CDC20,CDK1,Cyclin A,Cyclin B,Cyclin E,DHFR,DLGAP5,DLK1,E2f,E2F8,GMNN,HIST3H2A,IGFBP7,LAPTM4B,MAD2L1,MAK,NUSAP1,PBK,PLK4,PRC1,RACGAP1,Rb,RRM2,TCL1A,TFDP2,TFRC,TTK,TYMS,UBE2,UBE2C,UBE2S	48	26	Cell Death and Survival, Cancer, Organismal Injury and Abnormalities
2	ADAM8,B-cell receptor,BCL6,BCL11B,BPI,BTG3,CASP4,CCNB2,CD79A,CENPU,CKS2,CLEC10A,CLEC11A,HIP1,HLA-DR,IFN alpha/beta,Igg3,Ikb,IL-1R,IL13RA1,IL1R2,IL1RAP,KDM2A,KDM6B,NFkB (complex),OLFM4,PAX5,PGLYRP1,POU2AF1,SMC4,TH2 Cytokine,Tlr,TLR5,TNFRSF10C,TREM1	48	26	Hematological System Development and Function, Tissue Morphology, Cancer
3	ADRB,ARG1,ARL4A,AZU1,CaMKII,CENPF,COPS2,Creb,CREB5,DTL,ERMAP,H1F0,HIST1H1C,HIST1H4C,Histone H1,Histone h3,KCNJ2,KIAA0101,KIAA0226L,LEF1,MME,NRBF2,NREP,PCNA,Pkc(s),Rab5,Rac,RARA,RBX1,TCF,TLE3,TSEN34,UBE2D1,Ubiquitin,WLS	46	25	Gene Expression, Digestive System Development and Function, Embryonic Development
4	Alpha 1 antitrypsin,CAMP,CEACAM6,CEACAM8,chymotrypsin,CTSG,DEFA4,DEFA3 (includes others),Ecm,ELANE,elastase,ERK1/2,FCGR3A/FCGR3B,GK,GYPA,Hnp alpha,HP,ITGAX,LITAF,LTF,MMP25,MPO,Mucin,NUCB2,Par,PELI1,PI3,Pro-inflammatory	43	24	Infectious Diseases, Cell Death and Survival, Respiratory Disease

	Cytokine,PRTN3,SERPINB10,signal peptidase,TCN1,trypsin,UROD,XK			
5	Alpha tubulin,AQP1,BCR (complex),CA2,caspase,CD24,COX7B,CYB5A,cytochrome C,cytochrome-c oxidase,ERK,FABP5,FSCN1,GDF15,H2AFX,HIST1H2BC,HMMR,IgD,Igm,MAP2K1/2,MCL1,Mek,MIR101,NDUFA4,OSBPL10,Pak,PRDX2,Raf,RPS27L,SLC16A1,Sos,STMN1,TIP60,TOP2A,ZFP36L1	33	20	Molecular Transport, Cancer, Organismal Injury and Abnormalities
6	AKAP8,ATP8B4,BCAP29,C18orf25,CCDC8,CHTOP,CITED2,COPS5,CPNE3,CPOX,CREB3,CREB3L2,ELAVL1,FAM129A,FBXL5,GAS2L3,IL13,IL13RA1,IL1R2,ISCA1,LITAF,MAP3K7CL,MGST3,NCLN,NHLH1,PPOX,RBM47,SLC25A44,STX7,TMEM140,TMEM14B,TMEM30A,TOR1AIP1,ZNRF2,ZYG11B	27	17	Small Molecule Biochemistry, Gastrointestinal Disease, Hematological Disease
7	Actin,ADM,Cofilin,Collagen type I,Collagen(s),DOCK4,F Actin,GBP2,GCLM,Growth hormone,GYPB,GYPE,HBZ,hemoglobin,Il8r,Ldh (complex),LDL,LYN,MARCKS,MYB,NADPH oxidase,NAMPT,Notch,Pdgf (complex),PDGF BB,PI3K (complex),PI3K (family),PP2A,Rap1,RGCC,SNRPG,SORL1,SPTA1,STAT5a/b,TPM1	25	16	Cell Morphology, Organismal Injury and Abnormalities, Renal and Urological Disease
8	AES,AP1S2,AP2M1,AP5Z1,APP,ATG7,C4,CCNJL,CEACAM6,CMAS,CYTH4,DUSP11,DUT,ENY2,ESR1,GABARAP,GBA2,GTPase,HIST1H2BD,Iga,INIP,MIR22HG,MITF,MND1,NEDD4,RUNDC3A,SPC25,TECPR2,TMCC2,TNFAIP8L1,TP73,UBL3,UBXN10,UQCRB,UQCRQ	20	14	Neurological Disease, Developmental Disorder, Hereditary Disorder
9	Adaptor protein 2,ARHGAP26,CCR7,chemokine,chemokine receptor,Clathrin,CXCR1,CXCR2,F2RL1,FFAR2,Focal adhesion kinase,G protein alphai,Gi-coupled receptor,Gpcr,HCAR3,IgG1,IgG2b,IGHM,IL12 (family),Mapk,Metalloprotease,NMDA Receptor,NUMB,P110,p85 (pik3r),PTAFR,Ras,Ras homolog,RNASE2,S1PR1,SELPLG,Sfk,Shc,	19	13	Cellular Movement, Hematological System Development and Function, Immune Cell Trafficking

	SRC (family),tubulin (complex)			
10	26s Proteasome,Alp,ALPL,AMPK,CDKN2C,Ck2,collagen,DNAJB4,DUSP1,estrogen receptor,GATA3,HDL,HSP,Hsp70,Hsp90,HSPA6,Ifn,IFN Beta,IgG,IL1,Interferon alpha,KCNH2,LCN2,LOC254896,MHC Class II (complex),Mmp,Nos,P38 MAPK,POLR2K,RAD51AP1,RNA polymerase II,SECTM1,TAL1,Tnf (family),Vegf	19	13	Cancer, Gastrointestinal Disease, Organismal Injury and Abnormalities
11	ACSL1,Alpha catenin,Ap1,AQP9,BCL3,C/ebp,Collagen Alpha1,CYTIP,DAAM1,Fc gamma receptor,Fcer1,Fibrinogen,FNBP1L,Gm-csf,Ige,IgG2a,IL12 (complex),IL4R,Immunoglobulin,Jnk,KLHL2,MS4A3,N-cor,Nfat (family),Nr1h,PNP,Rar,RNASE3,Rxr,Smad 2/3,SYK/ZAP,Tgf beta,TGM2,THBS1,WIPF1	18	14	Cellular Function and Maintenance, Hematological System Development and Function, Post-Translational Modification
12	AKIRIN2,ANP32E,ASPRV1,CD276,CLEC5A,CYP1A1,DUSP11,EDEM3,FBXO31,HIST1H4B,HLA-J,HNF4A,HYI,iron,KCNK9,LSM5,LTB4R2,MAEA,MAN1C1,MARCH1,MINPP1,MTX3,NEK7,PRG2,REXO2,RHAG,RHCE/RHD,SMARCA4,TNF,TPP2,TRPC4AP,TSPO2,UCHL5,YIF1A,ZNF589	17	12	Cancer, Organismal Injury and Abnormalities, Gastrointestinal Disease
13	AURKA,Calmodulin,Cbp/p300,CD3,Cg,CHRNA5,Collagen type IV,FSH,glycolipid,Gsk3,HIST1H4J,HISTONE,Histone h4,ICAM4,Il31,Insulin,Integrin,KCNJ15,Lh,MCM4,NCAM2,Pka,PLC,PTX3,RAB13,RNU7-1,SCO1,SEC14L1,SEPP1,STOM,TCR,TUBB,TUBG1,Wnt,ZNFX1	16	12	Cancer, Organismal Injury and Abnormalities, Respiratory Disease
14	A1BG,CRISP3,ELF5,KNG1	1	1	Cancer, Gastrointestinal Disease, Hepatic System Disease

Haematological System Development & Function Network

Network 2 : Fluocinonide : PMFvsPVDrugSignatureActions : Fluocinonide

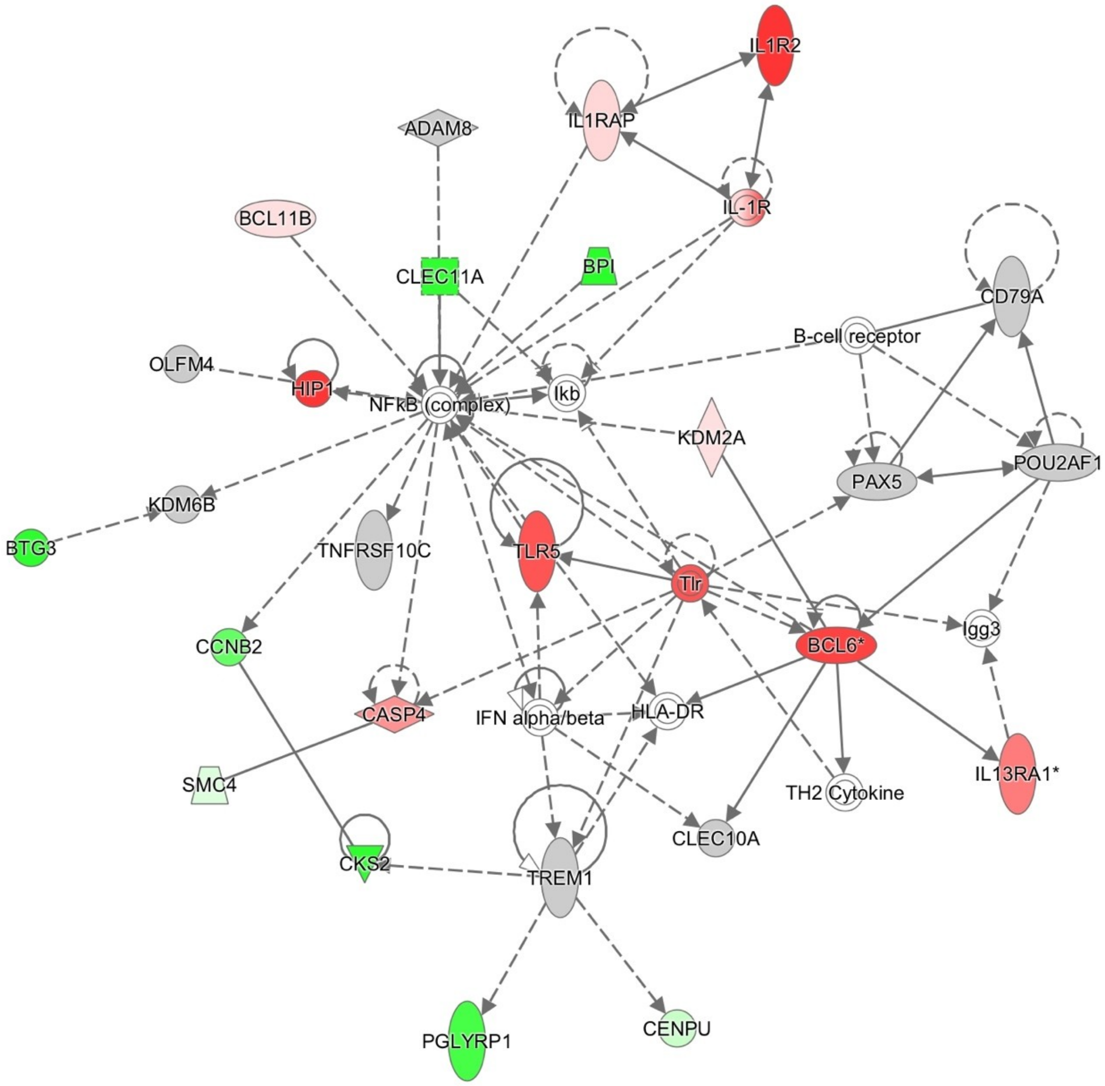

© 2000-2016 QIAGEN. All rights reserved.
